# Reducing burden of disease from residential indoor air exposures in Europe (HEALTHVENT project)

**DOI:** 10.1186/s12940-016-0101-8

**Published:** 2016-03-08

**Authors:** Arja Asikainen, Paolo Carrer, Stylianos Kephalopoulos, Eduardo de Oliveira Fernandes, Pawel Wargocki, Otto Hänninen

**Affiliations:** Department of Health Protection, National Institute for Health and Welfare, Neulaniementie 4, 70210 Kuopio, Finland; Department of Occupational Health, University of Milan, Via G.B. Grassi 74, 20157 Milan, Italy; European Commission, Joint Research Centre, Via Enrico Fermi 2749, I - 21027, Ispra, Italy; University of Porto, INEGI, Rua Dr. Roberto Frias, 4200 400 Porto, Portugal; Technical University of Denmark, International Centre for Indoor Environment and Energy, DTU Civil Engineering, 2800 Kps, Lyngsby, Denmark

## Abstract

**Background:**

The annual burden of disease caused indoor air pollution, including polluted outdoor air used to ventilate indoor spaces, is estimated to correspond to a loss of over 2 million healthy life years in the European Union (EU). Based on measurements of the European Environment Agency (EEA), approximately 90 % of EU citizens live in areas where the World Health Organization (WHO) guidelines for air quality of particulate matter sized < 2.5 mm (PM_2.5_) are not met. Since sources of pollution reside in both indoor and outdoor air, selecting the most appropriate ventilation strategy is not a simple and straightforward task.

**Methods:**

A framework for developing European health-based ventilation guidelines was created in 2010–2013 in the EU-funded HEALTHVENT project. As a part of the project, the potential efficiency of control policies to health effects caused by residential indoor exposures of fine particulate matter (PM_2.5_), outdoor bioaerosols, volatile organic compounds (VOC), carbon oxide (CO) radon and dampness was estimated. The analysis was based on scenario comparison, using an outdoor-indoor mass-balance model and varying the ventilation rates. Health effects were estimated with burden of diseases (BoD) calculations taking into account asthma, cardiovascular (CV) diseases, acute toxication, respiratory infections, lung cancer and chronic obstructive pulmonary disease (COPD).

**Results:**

The quantitative comparison of three main policy approaches, (i) optimising ventilation rates only; (ii) filtration of outdoor air; and (iii) indoor source control, showed that all three approaches are able to provide substantial reductions in the health risks, varying from approximately 20 % to 44 %, corresponding to 400 000 and 900 000 saved healthy life years in EU-26. PM_2.5_ caused majority of the health effects in all included countries, but the importance of the other pollutants varied by country.

**Conclusions:**

The present modelling shows, that combination of controlling the indoor air sources and selecting appropriate ventilation rate was the most effective to reduce health risks. If indoor sources cannot be removed or their emissions cannot be limited to an accepted level, ventilation needs to be increased to remove remaining pollutants. In these cases filtration of outdoor air may be needed to prevent increase of health risks.

**Electronic supplementary material:**

The online version of this article (doi:10.1186/s12940-016-0101-8) contains supplementary material, which is available to authorized users.

## Background

In the period 2006–2010, focus on indoor air quality has been raised by WHO, who has issued specific guidelines addressing air exposure in indoor spaces [1, 2]. Over the last previous two decades WHO had coordinated systematic reviews of scientific evidence and set Air Quality Guidelines [3, 4], although not specific for indoor air.

Requirements for indoor air quality (IAQ) in buildings is prescribed by existing standards for ventilation, but are often poorly related on health. At present, many ventilation standards (e.g. EN15251 [5]) define ventilation requirements in non-industrial buildings to meet comfort requirements of occupants, specified by the percentage of dissatisfied persons with indoor air quality and/or by the intensity of odour. While comfort is an important parameter, it does not fully reflect more serious health impacts like asthma, allergies, chronic obstructive pulmonary disease, cardiovascular diseases, lung cancer and acute toxication that are caused by exposures to pollutants present in indoor air. There are no European guidelines to recommend how the buildings should be ventilated to reduce the health risks of the occupants’ exposed to indoor air pollutants.

Direct scientific evidence on the relationship between ventilation and health is quite limited. In reviews by Wargocki et al. (2002) [6] and by Seppänen et al. (2004) [7], concentrating on office-type working environment and residential buildings, ventilation was considered to significant impact or association with comfort (perceived air quality, PAQ) and health (including sick building syndrome (SBS) symptoms, inflammation, infections, asthma, allergy, and short-term sick leaves), and productivity (performance of office work).

Li et al. (2007) [8] performed a systematic review of the role of the built environment in the transmission of airborne infectious agents. They concluded that there is strong evidence that suggests the association between ventilation, air movements in buildings and the transmission/spread of infectious diseases such as measles, tuberculosis, chickenpox, influenza, smallpox and severe acute respiratory syndrome (SARS).

Sundell et al. (2011) [9] identified 27 papers published in peer reviewed journals providing sufficient information on both ventilation rates and health effects in non-industrial buildings. Multiple health endpoints showed similar relationships with ventilation rate and were biologically plausible, although the literature did not provide clear evidence on particular agents. Higher ventilation rates in offices, up to about 25 l/s per person, were shown in the reviewed literature to be associated with reduced prevalence of SBS symptoms. Limited data suggested that inflammation, respiratory infections, asthma symptoms and short-term sick leave increase with lower ventilation rates. Home ventilation rates above 0.5 air changes per hour (h^−1^) were shown in the reviewed papers to be associated with reduced risk of allergic manifestations among children in a Nordic climate.

The role of outdoor air quality on indoor exposures was not addressed in any of the studies included in the above mentioned reviews, even though 90 % of EU citizens live in areas where the WHO guidelines for air quality for PM_2.5_ is not met [10]. Furthermore, the existence of indoor air sources was not systematically analysed or exposure levels quantified or considered when associating ventilation and health. Therefore, there is limited support from these previous studies on determining the best combination of source control and ventilation levels.

This work aims to summarize the current understanding of the sources of health risks in indoor environments and their relationship to ventilation requirements. Specific objectives of the study are: i) summarize the sources and magnitudes of health risks indoors, ii) provide a quantitative framework for estimation of long-term health effects caused by poor indoor air quality in residential buildings, and iii) quantify the burden of disease for each indoor exposure agent and evaluate efficiency of exposure reduction measures for EU-26 countries. In addition, the results are intended for development of national and international guidelines and standards, and can also be used as background information when analysing indoor air quality related issues in buildings.

## Methods

### Exposures

Ventilation plays a dual role in formation of indoor pollutant concentrations: on one hand it removes indoor generated pollutants from indoor spaces by ventilating the space with outdoor air, on the other hand, ventilation introduces outdoor air pollutants indoors [11, 12]. It has been shown that efficient filtering of intake air does not necessarily reduce penetration of outdoor air pollutants as much as could be expected based on the filtration efficiency as substantial fraction of the outdoor air enters indoors through windows, doors, ventilation ducts, and cracks and leaks in the building envelope [13].

When defining prevailing indoor air concentrations, a mass-balance model is needed to address the counter-acting roles of indoor and outdoor air sources. A commonly used approach based on Dockery and Spengler [14] and adopted in Hänninen et al. [11], [15] is as follows:

1 $$ \overline{C_i}=\frac{Pa}{a+k}\overline{C_a}+\frac{G}{V\left(a+k\right)}-\frac{\varDelta {C}_i}{\varDelta t\left(a+k\right)} $$

where *C*_*i*_ is the total indoor concentration (μg m^−3^) of the pollutant in question, *C*_*a*_ is the concentration in the intake air, *P* is the probability of the pollutant remaining suspended after penetrating through the building envelope, *α* is air exchange rate (h^−1^), *k* is the deposition rate of the pollutant indoors (h^−1^), *G* is the indoor generation level (μg h^−1^), *V* is the volume of the indoor space and *Δt* is change in temperature of the indoor space during sampling period (h). The third term covering the transient impacts of changing concentration can be considered zero when assuming steady-state for the sake of long-term average exposures.

Since the aim of this study is to estimate how changes in ventilation affect exposures in residential indoor environments, the probability distributions of national ventilation rates in residential building stock in year 2010 had to be estimated. Surprisingly limited data of measured ventilation rates are available for residences in European countries [16]. As a result, available measured data was reviewed and a regression model was developed combining the climatological and economical differences of European countries with ventilation rates. Further modelling with a Bayesian subjective probability approach was used for generation of lognormal probability distributions for ventilation rates in each EU-26 country (Table 1, list of countries and ventilation rate distributions, method described in detailed elsewhere [17]).

**Table 1:** Estimated ventilation rate distributions in European countries [17]

	Air exchange rate			Ventilation rate per occupant		
Country	Mean	Median	One-GSD	Mean	Median	One-GSD
			range^a^			range^a^
	h^−1^	h^−1^	h^−1^	lps pp	lps pp	lps pp
Austria	0.9	0.7	(0.4-1.3)	25	21	(11.1-39.1)
Belgium	0.7	0.6	(0.3-1.1)	17	14	(7.6-26.7)
Bulgaria	0.7	0.6	(0.3-1.1)	15	12	(6.4-22.3)
Cyprus	1.2	1.0	(0.5-1.9)	24	20	(10.6-37.2)
Czech Republic	0.6	0.5	(0.3-1.0)	14	11	(6.0-21.1)
Denmark	0.7	0.5	(0.3-1.0)	24	20	(10.4-36.6)
Estonia	0.7	0.5	(0.3-1.0)	13	10	(5.5-19.4)
Finland	0.7	0.5	(0.3-1.0)	17	14	(7.5-26.3)
France	0.6	0.5	(0.3-1.0)	18	14	(7.7-27.1)
Germany	0.7	0.6	(0.3-1.0)	20	17	(8.8-31.0)
Greece	1.0	0.8	(0.4-1.5)	20	17	(8.8-30.9)
Hungary	0.8	0.6	(0.3-1.2)	16	13	(6.8-24.0)
Ireland	0.6	0.5	(0.3-0.9)	14	12	(6.2-21.9)
Italy	0.8	0.6	(0.3-1.2)	21	17	(9.2-32.4)
Latvia	0.7	0.5	(0.3-1.0)	11	9	(4.9-17.2)
Lithuania	0.7	0.6	(0.3-1.0)	11	9	(4.9-17.3)
Luxembourg	0.9	0.7	(0.4-1.3)	32	26	(14.1-49.5)
Netherlands	0.7	0.6	(0.3-1.0)	21	17	(9.1-32.1)
Poland	0.7	0.6	(0.3-1.1)	11	9	(4.8-16.7)
Portugal	0.7	0.6	(0.3-1.1)	15	12	(6.6-23.1)
Romania	0.8	0.6	(0.3-1.2)	7	6	(3.2-11.1)
Slovakia	0.8	0.6	(0.3-1.2)	12	10	(5.1-17.9)
Slovenia	0.7	0.6	(0.3-1.1)	13	11	(5.9-20.7)
Spain	0.8	0.7	(0.3-1.2)	20	17	(8.9-31.3)
Sweden	0.6	0.5	(0.3-1.0)	20	17	(9.0-31.5)
UK	0.6	0.5	(0.3-0.9)	15	13	(6.8-23.8)
EU-26	0.7	0.6	(0.3-1.1)	17	14	(7.3-25.6)

^a^(median/GSD, median x GSD), GSD = Geometric Standard Deviation

### Risk model

A large number of indoor air pollutants have been associated with health responses, but some of those either play a small role for public health, or pose challenges for the exposure assessment or quantification of the burden of disease. Health determinants of housing in general are discussed in WHO 2011 [18], safe levels of specific chemicals indoors in WHO 2010 [2] and guidelines for exposure to dampness and mould specifically in WHO 2009 [1].

Exposures to environmental pollutants are associated with increased mortality and morbidity. Some of the widely used risk assessment methods estimate these separately as numbers of cases. The results from such incidence-based models are not comparable over different types of health endpoints and to improve comparability of impacts on various types of diseases and including mortality, disability adjusted life years (DALY) has been proposed as a common metric [19]. The following model and data used in this study and more details of calculations are presented in a technical report published earlier [20].

The burden of disease methodology makes the years of life lost (YLL) due to premature mortality and years lived with a disability (YLD) comparable and is summing them up as disability adjusted life years (DALY)

2 $$ DALY=YLL+YLD $$

The disabilities caused by various types of diseases are calculated accounting for the duration of the disease (*L*) and scaled using a disease specific disability weight (*DW*) and number of cases (N):

3 $$ YLD=DW\times L\times N $$

The current enhancement of the health impact assessment with the above described mass-balance approach, to account for varying ventilation, is built on the previous achievements of EnVIE [21] and IAIAQ projects [22] and the corresponding models for environmental burden of disease caused by indoor air quality. These models were based on pollutant specific attributable fraction (by expert judgements) of disease caused by indoor exposure and European level burden of disease (BoD) data. National estimates were produced by using national level BoD data and scaling the attributable fraction according to the ratio of national versus European indoor concentration estimates of each pollutant (i.e. PM_2.5_, outdoor bioaerosols, VOC, carbon oxide (CO) radon and dampness). Population weighted European average was used in cases where national level data was not available.

In the current work second hand smoke exposures at home were added to the list of pollutants using exposure data from a European survey [23]. In addition, the earlier PM_2.5_, radon and dampness models were updated to the relative risk-based population attributable fraction (*PAF*) as [24]:

4 $$ PAF=\frac{f\times \left(RR-1\right)}{f\times \left(RR-1\right)+1} $$

where *f* is the fraction of population exposed to a given factor and *RR* is the relative risk of the exposed population. WHO estimates for national burden of disease in 2004 were used for the background BoD [25] (see supporting information, Additional file 1: Table S1) to calculate the environmental burden of disease (EBD) caused by the current exposures (Table 2)

**Table 2:** Outdoor and indoor exposure levels (PM_2.5_, radon and VOC) and prevalence of exposure (dampness in homes and second hand smoke of non-smoking population) in European countries used for burden of disease calculations

	^a)^Out. PM_2.5_	^b)^Ind. PM_2.5_	^c)^Out. VOC	^d)^Ind. VOC	^e)^Ind. Radon	^f)^Dampness homes	^g)^SHS non-smokers
	μg m^−3^	μg m^−3^	μg m^−3^	μg m^−3^	Bq m^−3^	%	%
Austria	17	5	*103*	*298*	97	8	14
Belgium	19	5	*103*	*298*	69	14	18
Bulgaria	22	5	*103*	*298*	30	n/a	23
Cyprus	23	4	*103*	*298*	7	30	31
Czech Republic	23	5	116	334 ^(8^	140	16	16
Denmark	13	3	*103*	*298*	53	11	17
Estonia	11	3	*103*	*298*	120	23	16
Finland	9	3	64	226 ^(8^	120	5	2
France	12	5	77	223 ^(9^	89	14	9
Germany	16	5	*103*	297 ^*(10*^	50	13	13
Greece	21	4	155	345 ^(9^	55	19	28
Hungary	25	5	*103*	*298*	107	19	12
Ireland	8	3	*103*	*298*	89	15	14
Italy	20	4	181	489 ^(8^	70	21	11
Latvia	12	3	*103*	*298*	n/a	26	12
Lithuania	14	3	*103*	*298*	55	25	28
Luxembourg	12	5	52	148 ^(9^	115	15	8
Netherlands	19	5	46	134 ^(9^	30	18	15
Poland	22	5	*103*	*298*	49	37	21
Portugal	18	4	38	213 ^(9^	86	20	13
Romania	23	5	*103*	*298*	45	29	23
Slovakia	23	5	*103*	*298*	87	6	13
Slovenia	17	5	*103*	*298*	87	17	14
Spain	16	4	*103*	*298*	90	18	20
Sweden	10	3	77	223 ^(11^	108	6	3
UK	13	3	85	245 ^(8^	20	15	7
EU-26	17	4	104	298	64	18	14

^a)^De Leeuw and Horalek, 2009 [28], ^b)^Hänninen, et al. 2004 [11] (in case of missing data, European average value used), ^c)^Outdoor VOC concentrations were calculated based on indoor concentrations by using data on fraction estimations from literature (Finland 0,22, Greece 0.31, Italy 0.27, Portugal 0.15, other countries 0.26 (population weighted EU-26 mean value based on the four previous values), ^d)^Data on indoor VOC concentrations collected from several source (indicated separately, in case of missing data, European average value used, indicated with *italics*), ^e)^EU Radonmapping [29] (in case of missing data, European average value used), ^f)^WHO/ENHIS Fact Sheet 3.5 [30], ^g)^Survey on Tobacco, Eurobarometer 253, 2009 [23], ^h)^EXPOLIS study Jantunen MJ. et al. 1998 [31], ^i)^IAIAQ model, Jantunen et al. 2012 [22], ^j)^GerEs study: http://www.umweltbundesamt.de/en/topics/health/assessing-environmentally-related-health-risks/german-environmental-survey-geres, ^k)^Norback, D. et al. 1995 [32].

5 $$ EBD=PAF\times BoD $$

The relative risk at the current exposure level (E) was estimated from epidemiological relative risk (*RR°*) expressed per a standard exposure increment:

6 $$ RR={e}^{\left(E \ln RR{}^{\circ}\right)}=RR{{}^{\circ}}^E $$

Pollutant specific diseases and methodology are presented in Table 3. PAFs for each country and pollutant can be found from supporting information Additional file 2: Table S2.

**Table 3:** Diseases and exposure-response relationships included in this assessment

Exposures^a^	Health endpoints	WHO	RR	PAF	RR & PAF source(s)	BoD calculation^b^
PM_2.5_	Asthma	W113	1.009	f(RR, E)^c^	Pope et al. 2002 [33]	PAF(E, RR) × BoD_2004_
	Lung cancer	W067	1.014	f(RR, E)^c^	Pope et al. 2002 [33]	PAF(E, RR) × BoD_2004_
	CV-diseases	W104	1.009	f(RR, E)^c^	Pope et al. 2002 [33]	PAF(E, RR) × BoD_2004_
	COPD	W112	1.009	f(RR, E)^c^	Pope et al. 2002 [33]	PAF(E, RR) × BoD_2004_
Outdoor bioaerosols	Asthma	W113	n/a	0.1^d^	Jantunen et al., 2010 [22]	PAF × BoD_2004_
VOC	Asthma	W113	n/a	0.05^e^	Jantunen et al., 2010 [22]	C/C_EU_ × PAF × BoD_2004_
CO	Acute toxication caused by carbon monoxide	n/a	n/a	0.9^e^	Jantunen et al., 2010 [22]	Cases x 20 years lost/case
Radon	Lung cancer	W067	1.0014	f(RR, E)^c^	Darby et al., 2005 [34]	PAF(E, RR) × BoD_2004_
Home dampness	Respiratory infections	W038	1.37	f(RR, E)^c^	Fisk et al., 2007 [35]	PAF(E, RR) × BoD_2004_
	Asthma	W113	1.5	f(RR, E)^c^	Fisk et al., 2007 [35]	PAF(E, RR) × BoD_2004_
SHS^f^	Lung cancer	W067	1.21	f(RR, E)^c^	US S.G. 2006 [36]	PAF(E, RR) × BoD_2004_
	Ischaemic heart disease	W107	1.27	f(RR, E)^c^	US S.G. 2006 [36]	PAF(E, RR) × BoD_2004_
	Asthma	W113	1.97	f(RR, E)^c^	Jaakkola et al., 2003 [37]	PAF(E, RR) × BoD_2004_

^a)^Population weighted average in EU26, ^b)^C = National population weighted concentration, CEU = European average concentration, E = National population weighted exposure, ^c)^Calculated as PAF = (f × (RR-1))/((f × (RR-1)) + 1), where RR = RR°E (see equation 4) [24], ^d)^Original value of 0.25 in Jantunen et al. (2010) [22] adjusted to 0.1 due to separation of indoor and outdoor sources and focusing on pollen from outdoor air, ^e)^Expert judgment PAF from the EnVIE panel used directly [21], see column PAF,

^f)^Second hand smoke exposure of non-smoking adults at home.

### Exposure control scenarios

The mass-balance enhanced burden of disease model was used to evaluate the ability of three alternative exposure control scenarios to reduce BoD. In the first scenario the ventilation level was optimised by finding the ventilation rate with lowest health effects (i.e. solving the health-based optimum level of ventilation). In the second scenario optimised ventilation was combined with adjusting the filtration level to control outdoor pollutants entering indoors. In the third scenario indoor sources were controlled with ventilation set to the level of minimum requirements.

#### Scenario 1: Dilution with health-based optimum level ventilation

In the first exposure reduction scenario, indoor and outdoor sources were kept unchanged and only ventilation rate was adjusted to find the health-based optimum level of ventilation. In this scenario, increased level of ventilation decreases the pollutant concentrations from indoor sources and increases contribution of outdoor sources indoors. The health-based optimum level of ventilation was solved for each country among EU-26 by calculating the burden of disease with ventilation rates from 0.1 to 50 lps pp.

Use of mass-balance dilution model is based on assumption that all indoor originating sources follow a constant rate of emissions even though this is not self-evident for several indoor originating pollutants, in this case especially for radon and dampness. In some ventilation systems higher ventilation rates may cause under pressure indoors and this may increase infiltration of radon from the soil below the buildings. In addition, in cases where dampness is created by condensation, higher ventilation rates may increase the problem. However, the benefits of higher ventilation rates were calculated assuming the mass-balance for a constant source term for these pollutants too.

#### Scenario 2: Filtration of intake air

Analyses of scenario 1 showed that in cases where outdoor air is polluted, it may become a significant source of exposures indoors. Therefore, in the second scenario the burden of disease was attempted to reduce by controlling the penetration of pollutants from outdoors to indoors with increased filtration. Filtration efficiency was specified for PM_2.5_ particles.

Three levels of filtration were compared. In the baseline scenario it was assumed that 90 % of the outdoor PM_2.5_ mass concentration penetrates indoors. In addition, realistic but increasingly challenging penetration levels of 70 % and 50 % were evaluated. These correspond to effective filtration of PM_2.5_ mass concentration by 27 % and 45 %, respectively, and these kind of filtration levels can be achieved in real buildings at least when using mechanical ventilation systems [13]. When discussing the filtration efficiencies of filters and the above mentioned penetration efficiencies, it has to be noted that the penetration efficiency is defined for the building, accounting for leaks and ventilation from windows, doors etc.

The ventilation rate in this scenario was set to a health-based optimum ventilation (i.e. ventilation level with the lowest health effects) defined separately for each filtration level. Burden of disease was then calculated with these ventilation levels and compared to the BoD in the baseline scenario to estimate the achievable health benefits.

#### Scenario 3: Source control and minimum ventilation (4 lps pp)

In the third scenario the exposures of the indoor sources were assumed to be reduced with the following emission control potentials for the considered pollutants:

-90 % for radon, carbon monoxide (CO) and second hand smoke (SHS)
-50 % for volatile organic compounds (VOC) and dampness
-25 % for particulate matter (PM_2.5_)

These control potentials were defined as hypothetical, technically feasible maximum reductions evaluated by the authors. The radon estimate assumes highly efficient application and control of radon safe construction in radon-prone areas combined with control of second hand smoke exposures known to act synergistically with radon. Efficient second hand smoke reductions have already been demonstrated in Finland in both workplaces and in homes resulting a decrease in proportion of adolescents exposed to SHS from 17 % in 1991 to 6 % in 2009 [26] and the SHS policies are moving forward also on at European level. The carbon monoxide exposures were assumed to be reduced by compulsory alarms that will notify residents when carbon monoxide levels are increasing because of malfunctioning devices or fires.

VOC levels are assumed to be controlled by new low emitting products and wider use of comprehensive labelling systems for low emission products. Dampness controls need to combine better constructions practises and structural improvements with active/online and passive warning sensors. However, the greatest challenge is to control particulate matter. The proposed 25 % reduction is assumed to be achieved with target exhausts in kitchens, avoiding use of candles and improved design of combustion devices.

To provide some sensitivity analysis to estimate the effectiveness of source control, two other scenarios with lower and higher source control capabilities were also analysed. In the lower source control scenario (scenario 3.1), it was assumed a reduction of 80 % for radon, CO and SHS and 25 % reduction of PM_2.5_, VOC and dampness exposures. In the higher source control scenario (scenario 3.2), a total control (100 %) of radon, CO and SHS and 75 % reduction of PM_2.5_, VOC and dampness exposures were assumed.

In all source control scenarios, the ventilation level was set to be 4 lps pp, which was proposed as base ventilation rate in cases when ventilation must handle only human bio-effluent emissions (carbon dioxide (CO_2_) and moisture) by work done in HealthVent (report will be published separately). In presence of any other indoor sources, level of ventilation needs to be adjusted to acceptable level.

## Results

### Burden of disease caused by indoor exposures in 2010

An annual loss of 2.1 million DALYs in EU-26 is associated to indoor and outdoor originating pollutants with more than half of it (1.28 million DALYs) caused by exposures to outdoor air pollution indoors and the remaining 0.74 million DALYs caused by indoor source pollutants.

**Fig. 1:**
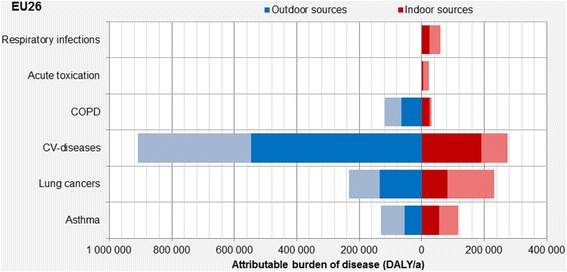
Attributable burden of diseases due to indoor exposures in 2010 in EU-26. The lighter shade of blue and red represents the maximum fraction that can be reduced by actions (scenarios) presented in this paper (reproduced from Asikainen & Hänninen [20])

This burden of disease is dominated by cardiovascular (CV) diseases as a result of exposure to outdoor and indoor particles and second hand smoke, corresponding 57 % of the total burden of disease (Fig. 1). The second largest contribution comes from lung cancer (23 %) and the third in the list is asthma (total of 12 %). The remaining 8 % is divided between various respiratory symptoms and conditions.

**Fig. 2:**
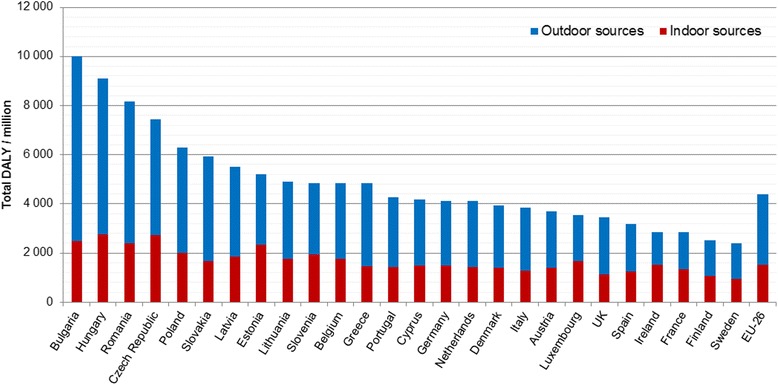
Total burden of disease as DALY/million population from indoor exposures in EU-26 countries with division to indoor and outdoor sources in the 2010 building stock (reproduced from Asikainen & Hänninen [20])

The total burden of disease for individual countries varies considerably with the highest burden of 10 000 DALY per one million population in Bulgaria to the lowest one of 2 000 DALY per million in Sweden. The EU-26 average burden of disease is slightly over 4 000 DALY in a year per one million population. The higher levels in East-European countries are dominated by high contribution of outdoor sources, which vary from 46 % (Ireland) to 75 % (Bulgaria) (Fig. 2).

### Source contributions to burden of disease

**Fig. 3:**
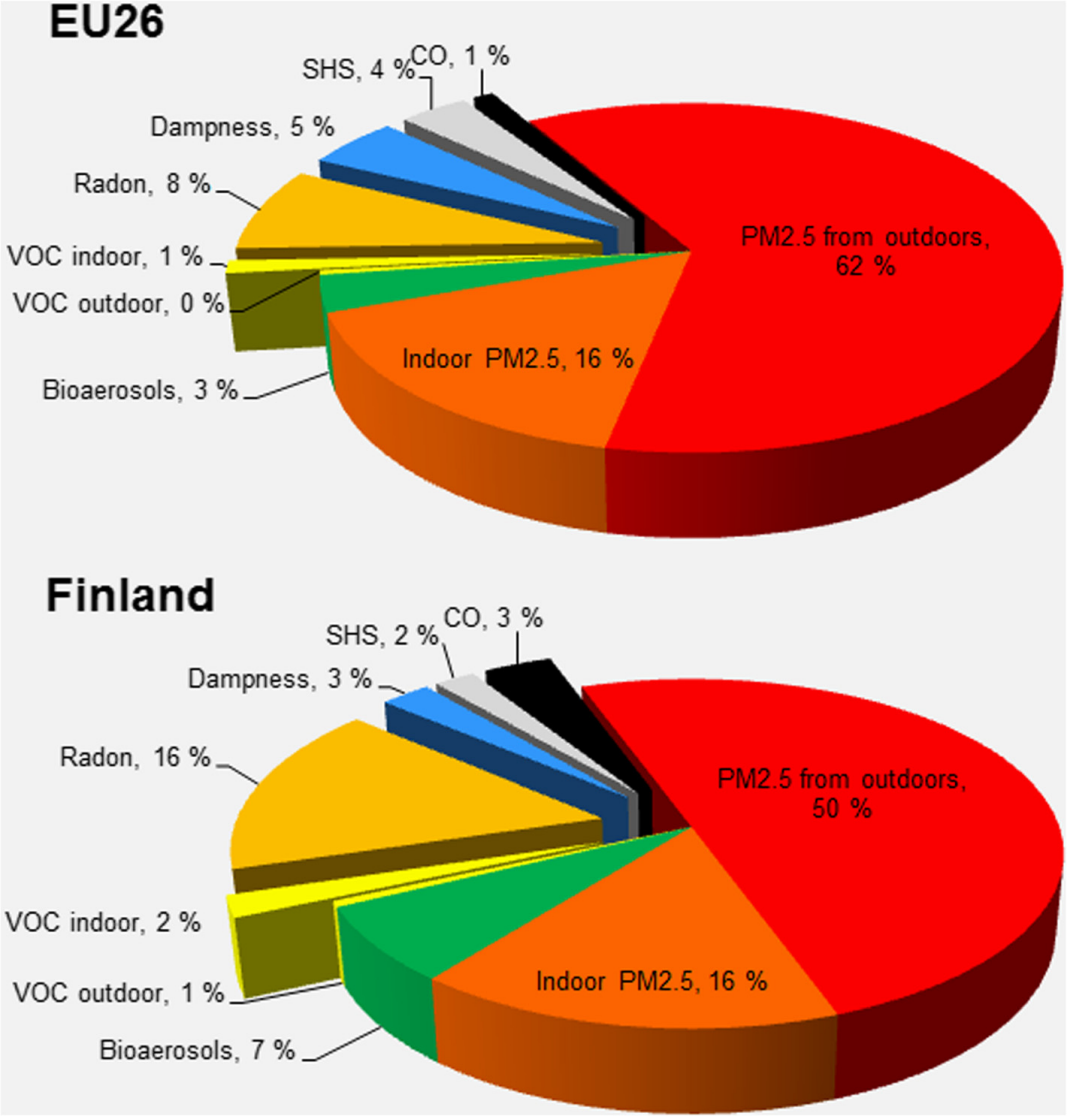
Burden of disease attributable to indoor exposures in EU-26 (2.1 M DALY/a) and in Finland (13 k DALY/a) in 2010 divided into source contributions (reproduced from Asikainen & Hänninen [20])

Overall in EU-26, almost 80 % of the total annual burden of disease of indoor exposures (i.e. 4 000 DALYs/million) is estimated to be caused by PM_2.5_, dominated by particulates originating from outdoor air penetrating indoors (Fig. 3).

Comparison of the sources of burden of disease in Finland (Fig. 3) and in other EU-26 countries (Table 4) shows clear differences between countries. As an example, in Finland the role of ambient particles is lower than on average in the EU-26 countries, but both bio-aerosols (pollen) and radon play much more significant roles, the latter contributing double to that than for the European average.

**Table 4:** Contribution (%) of different sources to the total DALYs in 2010

Country	Ind. PM_2.5_	Radon	Ind. VOC	CO	Damp.	SHS	Out. PM_2.5_	Bio-aerosols	Out. VOC
Austria	18	11	1	1	1	5	58	3	0
Belgium	18	9	1	0	4	4	60	3	0
Bulgaria	17	2	0	0	3	2	74	1	0
Cyprus	11	0	1	0	11	12	61	3	0
Czech Republic	14	12	1	4	3	3	61	2	0
Denmark	13	9	2	3	3	6	59	5	1
Estonia	14	13	1	5	8	4	52	3	0
Finland	16	16	2	4	3	2	50	7	1
France	20	18	2	0	5	2	46	6	1
Germany	20	6	1	1	3	4	60	3	0
Greece	14	6	1	1	4	5	68	2	0
Hungary	15	12	0	1	1	1	69	1	0
Ireland	13	12	4	2	11	12	34	11	1
Italy	14	9	2	1	4	3	64	3	1
Latvia	15	6	1	2	7	3	64	2	0
Lithuania	14	5	0	0	6	10	63	1	0
Luxembourg	21	15	1	1	6	3	47	5	0
Netherlands	18	4	1	1	6	5	61	5	0
Poland	15	5	1	1	6	3	66	2	0
Portugal	14	7	1	1	7	4	62	4	0
Romania	16	3	0	1	7	2	70	1	0
Slovakia	16	7	1	1	2	3	70	2	0
Slovenia	17	11	1	2	5	3	56	3	0
Spain	14	14	1	0	5	4	57	4	0
Sweden	16	15	2	2	3	3	54	6	1
United	13	3	2	1	8	5	59	8	1
EU-26	16	8	1	1	5	4	62	3	0

Even though dampness and mould problems continuously raise a lot of attention in Finland, only 3 % of the total BoD is estimated to be caused by dampness being at the lower end on the European scale (ranging from 1 % to 11 %),

### Health benefits of exposure control scenarios

**Fig. 4:**
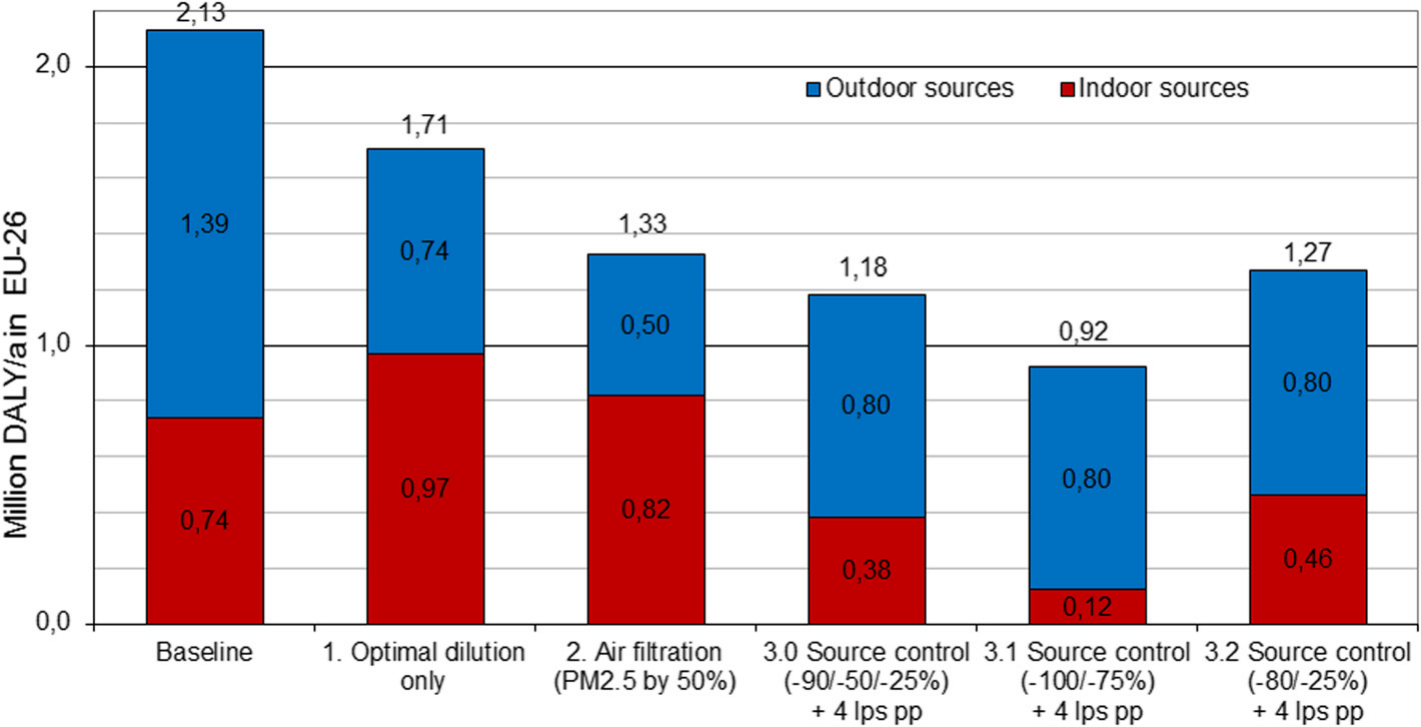
Burden of disease at the baseline (2010) in comparison with alternative potential control strategies in EU-26 (in millions of DALYs) (reproduced from Asikainen & Hänninen [20])

Each of the described three exposure control scenarios provides health benefits compared to the 2010 baseline scenario in EU-26 so, that 20 % reduction of burden of disease was achieved with the dilution scenario, 38 % reduction with the filtration scenario, and 44 % reduction with the indoor source control scenario (changing from 41 % to 54 % depending on assumed source reductions) (Fig. 4).

In the dilution-based scenario 1, reduction of indoor originating exposures with increased ventilation is compensated by increased penetration of outdoor pollution leading to a lower health benefits. In this scenario, the lowest burden of disease in EU-26 is found at mean ventilation level of 4.4 lps pp, which is clearly lower than the mean ventilation in the existing building stock (17 lps pp) defined by the regression modelled probability distributions (Table 1) [17, 20].

Approximately twice as high benefits are achievable by filtration of outdoor air in scenario 2. The results for maximum feasible filtration (with penetration fraction P = 50 %) show that 38 % reduction in burden of disease (800 000 DALYs) in EU-26 can be achieved. The European optimum mean ventilation level with lowest burden of disease is then 7.7 lps pp. This scenario would in practice imply substantial change towards mechanical ventilation systems in Europe. In the Nordic countries, this is already the practice due to the energy efficiency norms, but, in the majority of the European building stock, the filtration scenario would require a substantial step towards installing mechanical systems.

The largest health benefits can be achieved by the source control approach (scenario 3.0), which significantly reduces the need to control exposures by dilution. The benefits are approximately 44 % from the baseline (940 000 DALYs) in EU-26, and changing from reduction of 41 % (865 000 DALYs) with lower source control assumptions in scenario 3.1to 57 % (1.21 million DALY) with higher source control assumptions in scenario 3.2, demonstrating source control to be more effective than dilution or filtration even with smaller reductions of source exposures.

In addition to higher health benefits and compared to the filtration-based scenario 2, the advantage of the source control scenario 3 is that lower dilution need (i.e. enabling lower ventilation rate) allows also for lower infiltration of outdoor particles and therefore the feasibility of approach is better in the current building stock. Moreover, requiring lower ventilation rates the source control approach is likely to prove also more energy-efficient.

Further analysis of the contribution of indoor and outdoor sources shows, that with the dilution scenario 1 the health benefits are not only due to smaller proportion of the indoor contribution (i.e. the dilution of the pollutants from the indoor sources), but is mainly due to the lower ventilation rates that limit the penetration of the outdoor pollutants indoors.

In the filtration scenario 2 the health benefits are due to filtration of the outdoor pollutants and also effective dilution of the indoor pollutants, as the health-based optimal ventilation levels are higher.

Furthermore, in the source control scenarios, the health benefits are a result of both effects: lower indoor sources due to the source control and lower penetration of outdoor pollutants due to low level of ventilation.

## Discussion

The results suggest that (i) there is a substantial burden of disease associated with exposures taking place indoors and that (ii) these risks can substantially be reduced by range of control actions affecting indoor pollution sources, infiltration of outdoor pollutants, and ventilation levels. Besides the estimated health benefits and policy implementation costs, the suggested prioritization of the policies depends also on the uncertainties of the estimates.

Substantial uncertainty is raised by model uncertainties, including the selection of pollutants to be considered as relevant for indoor exposures and the health end-points associated with them. It is not clear how much the burden of disease estimates are underestimated due to the dozens of ignored exposures or missing health endpoints for the included exposures. The model uncertainties could and should be qualitatively evaluated by experts before the implementation of results.

The other aspect of the model uncertainties is related on model parameters. Variable degree of uncertainty exists in the exposure-response response relationships based on epidemiological studies. For some of the included pollutants, such as PM_2.5_ originating from outdoor air, this data is based on a large number of studies, representing very large populations in different climatological regions. In this study, the exposure-response relationship of ambient particles has also been used for indoor generated particles. The indoor generated particles have partly similar composition, originating from combustion processes or being re-suspended particles originating from soil, thus it is reasonable to assume similar toxicity as for the ambient particles. However, some particle fractions, especially the particles generated from food cooking, from the skin and clothes of occupants, and from interior textiles, have a different chemical composition with limited direct evidence on their toxicity. This may lead on overestimation of the health effects caused by indoor PM_2.5_. Significance of this varies between the countries, since portion of health effects caused by indoor PM_2.5_ from the total burden of disease on baseline 2010 changes from 13 % to 21 % (Table 4). Average contribution of indoor PM_2.5_ in EU-26 is 16 % (Fig. 3), so this fraction could be removed from total BoD, if considered too uncertain due to the lack of toxicological evidence. In this case, the contribution of indoor sources would decrease in all scenarios and outdoor component would become the dominant one, also in the scenario 2 and in the scenario 3. However, the overall performance and order of scenarios would not change, so the conclusions would remain the same.

Scenarios including projections to future rely always on assumed changes in the investigated environments and factors, and we may not know all changes that need to be accounted for. Also the implementation timeframe of selected actions or policies need to be considered. In this case the most significant element in the scenario uncertainties is related to the development of future building stocks [27]. This includes also changes in the technical solutions of ventilation systems, which are not specifically defined in the current ventilation guidelines. An example of this is the filtration of outdoor air pollution, especially PM_2.5_, but also pollen, other biological particles, ozone, ultrafine traffic particles and so on. Infiltration of ambient particles depends on air exchange rates, size distribution of the outdoor particles, and filtration of the intake air. At lower air exchange rates the prolonged residence time of air indoors and corresponding deposition of the larger particles on indoor surfaces reduces indoor exposures even when the outdoor air is not filtrated. Using window frames and other sedimentation chambers allows for filtrating particles even in natural ventilation systems. Nevertheless, active filtration becomes efficient only in mechanical systems using high quality (above FP7) filters.

The used ventilation rate estimates per occupant (lps pp) are calculated using average residence sizes and average numbers of occupants in each country. Population weighted average outdoor concentrations have also been used to estimate the indoor exposures. It is clear that the air filtration needs for a specific building have to be defined taken into account the ambient air quality at the selected building location. In all countries considered, there are locations where the outdoor levels exceed the WHO guidelines much more than the national averages used here indicate. When the current methods are proposed to determine the potential filtration needs, they have to be applied with worst case estimates for the actual building site, accounting for the whole service life cycle.

Largest health benefits were projected for the source control policies. It is obvious that the benefits are achievable only if the source controls work as efficiently as proposed and that the efficiency of the source controls must be confirmed with follow-up (e.g. auditing) of exposure levels after the policy enforcement.

## Conclusions

Over 2 million disability adjusted life years (DALY) are annually lost in the European Union due to compromised indoor air quality, but this burden of disease can be reduced by adjusting ventilation, filtration of intake air and by controlling indoor sources. All three approaches are able to provide substantial reductions in the health risks from approximately 20 % to almost 50 %, corresponding to 400 000 and 900 000 saved DALYs in EU-26. Thus selection of strategies has substantial impact on the expected benefits.

The projected health benefits can be achieved if the controls on ventilation and sources are fully implemented as defined in the scenario descriptions. In the case of selecting some of the proposed strategies for implementation, a careful follow-up plan has to be developed for ensuring that the controls are effective and match the requirements of the benefit calculations.

## Additional files

Additional file 1: Table S1. National background burden of disease (BoD) in DALY/100 000 (WHO 2004). Data table provides background burden of disease values for each 26 EU countries included in the model used in this paper. (DOCX 16 kb) Additional file 2: Table S2. National population attributable fractions (PAFs) with baseline 2010 exposure concentrations. Data table provides population attributable fractions (i.e. fraction of disease caused by pollutant specific exposure) for 2010 baseline situation for each 26 EU countries included in the model used in this paper. (DOCX 16 kb) Additional file 3:
**Peer review reports.** (PDF 724 kb)

## Abbreviations

BoD: burden of disease
CO: carbon monoxide
CO2: carbon dioxide
CV: cardiovascular
DALY: disability adjusted life years
DW: disability weight
EBD: environmental burden of disease
EU: European Union
IAQ: indoor air quality
L: duration of the disease
lps pp: liters per second per person
PAF: population attributable fraction
PAQ: perceived air quality
PM_2.5_: particulate matter sized < 2.5 mm
RR: relative risk
SARS: severe acute respiratory syndrome
SBS: sick building syndrome
SHS: second hand smoke
VOC: volatile organic compounds
WHO: World Health Organization
YLD: years lived with a disability
YLL: years of life lost
