# Supplementary material for: Reducing burden of disease from residential indoor air exposures in Europe (HEALTHVENT project)
Source: Environ Health. 2016 Mar 8;15(Suppl 1):35. doi: 10.1186/s12940-016-0101-8 (PMC4895703; doi:10.1186/s12940-016-0101-8)
Supplement: Additional file 1: Table S1. — National background burden of disease (BoD) in DALY/100 000 (WHO 2004). Data table provides background burden of disease values for each 26 EU countries included in the model used in this paper. (DOCX 16 kb) [file 12940_2016_101_MOESM1_ESM.docx]

Supporting information, Table 1a. National background burden of disease (BoD) in DALY/100 000 (WHO 2004).

| **Country** | **Total BoD** | **Asthma** | **Lung (trachea & bronchus) cancer** | **CV-disease** | **Chronic obstructive pulmonary disease** | **Upper and lower respiratory disease symptoms** | **Acute CO toxication ^(1^** |
| --- | --- | --- | --- | --- | --- | --- | --- |
| **Austria** | 12 069 | 129 | 349 | 1 828 | 415 | 74 | 2 |
| **Belgium** | 12 948 | 150 | 515 | 2 129 | 597 | 147 | 2 |
| **Bulgaria** | 18 296 | 93 | 416 | 6 924 | 181 | 245 | 5 |
| **Cyprus** | 12 010 | 122 | 178 | 2 258 | 25 | 247 | 1 |
| **Czech Republic** | 14 326 | 160 | 495 | 3 358 | 350 | 165 | 34 |
| **Denmark** | 13 447 | 178 | 502 | 2 093 | 977 | 86 | 14 |
| **Estonia** | 18 900 | 168 | 435 | 4 676 | 163 | 260 | 27 |
| **Finland** | 13 205 | 178 | 264 | 2 305 | 265 | 136 | 10 |
| **France** | 12 262 | 173 | 446 | 1 415 | 178 | 88 | 0 |
| **Germany** | 12 536 | 137 | 407 | 2 392 | 428 | 108 | 6 |
| **Greece** | 11 826 | 86 | 437 | 2 764 | 162 | 157 | 5 |
| **Hungary** | 17 941 | 72 | 787 | 4 193 | 542 | 96 | 12 |
| **Ireland** | 11 692 | 307 | 309 | 1 735 | 382 | 211 | 5 |
| **Italy** | 11 245 | 108 | 406 | 1 941 | 256 | 76 | 5 |
| **Latvia** | 19 615 | 115 | 397 | 5 705 | 131 | 246 | 11 |
| **Lithuania** | 18 401 | 66 | 357 | 4 319 | 310 | 216 | 2 |
| **Luxembourg** | 12 341 | 175 | 378 | 2 002 | 416 | 162 | 4 |
| **Netherlands** | 11 486 | 196 | 486 | 1 707 | 460 | 143 | 5 |
| **Poland** | 14 911 | 136 | 541 | 3 245 | 164 | 148 | 5 |
| **Portugal** | 13 615 | 169 | 284 | 2 416 | 357 | 189 | 5 |
| **Romania** | 17 685 | 61 | 403 | 5 009 | 269 | 420 | 5 |
| **Slovakia** | 15 340 | 104 | 358 | 3 422 | 162 | 256 | 3 |
| **Slovenia** | 14 002 | 164 | 497 | 2 464 | 353 | 208 | 12 |
| **Spain** | 11 352 | 121 | 384 | 1 556 | 311 | 85 | 1 |
| **Sweden** | 11 478 | 141 | 255 | 2 004 | 329 | 117 | 4 |
| **UK** | 12 871 | 263 | 394 | 2 083 | 545 | 216 | 5 |
| **Europe-26** | 13 055 | 150 | 426 | 2 432 | 341 | 143 | 5 |

^1)^ Calculated based on unpublished national data on CO toxications. For missing data the population weighted avarages used.
