# Supplementary material for: Reducing burden of disease from residential indoor air exposures in Europe (HEALTHVENT project)
Source: Environ Health. 2016 Mar 8;15(Suppl 1):35. doi: 10.1186/s12940-016-0101-8 (PMC4895703; doi:10.1186/s12940-016-0101-8)
Supplement: Additional file 2: Table S2. — National population attributable fractions (PAFs) with baseline 2010 exposure concentrations. Data table provides population attributable fractions (i.e. fraction of disease caused by pollutant specific exposure) for 2010 baseline situation for each 26 EU countries included in the model used in this paper. (DOCX 16 kb) [file 12940_2016_101_MOESM2_ESM.docx]

Supporting information, Table 2a. National population attributable fractions (PAFs) with baseline 2010 exposure concentrations.

|  | **Radon** | **PM_2.5_** | | | | **Dampness** | |
| --- | --- | --- | --- | --- | --- | --- | --- |
| **Country** | **Lung cancer** | **Cardiovascular diseases, indoor** | **Lung cancer, indoor** | **Cardiovascular diseases, outdoor** | **Lung cancer, outdoor** | **Astma** | **Upper and lower respiratory symptoms** |
| **Austria** | 0.03 | 0.02 | 0.03 | 0.07 | 0.11 | 0.03 | 0.04 |
| **Belgium** | 0.02 | 0.02 | 0.03 | 0.08 | 0.12 | 0.05 | 0.07 |
| **Bulgaria** | 0.01 | 0.02 | 0.03 | 0.09 | 0.14 | 0.06 | 0.08 |
| **Cyprus** | 0.00 | 0.01 | 0.02 | 0.10 | 0.15 | 0.10 | 0.13 |
| **Czech Republic** | 0.04 | 0.02 | 0.03 | 0.10 | 0.15 | 0.06 | 0.07 |
| **Denmark** | 0.01 | 0.01 | 0.02 | 0.06 | 0.09 | 0.04 | 0.05 |
| **Estonia** | 0.03 | 0.01 | 0.02 | 0.05 | 0.07 | 0.08 | 0.10 |
| **Finland** | 0.03 | 0.01 | 0.02 | 0.04 | 0.06 | 0.02 | 0.02 |
| **France** | 0.02 | 0.02 | 0.03 | 0.05 | 0.08 | 0.05 | 0.07 |
| **Germany** | 0.01 | 0.02 | 0.03 | 0.07 | 0.11 | 0.05 | 0.06 |
| **Greece** | 0.02 | 0.01 | 0.02 | 0.09 | 0.13 | 0.07 | 0.09 |
| **Hungary** | 0.03 | 0.02 | 0.03 | 0.10 | 0.16 | 0.07 | 0.09 |
| **Ireland** | 0.02 | 0.01 | 0.02 | 0.03 | 0.05 | 0.05 | 0.07 |
| **Italy** | 0.02 | 0.01 | 0.02 | 0.08 | 0.13 | 0.07 | 0.10 |
| **Latvia** | 0.02 | 0.01 | 0.02 | 0.05 | 0.08 | 0.09 | 0.12 |
| **Lithuania** | 0.02 | 0.01 | 0.02 | 0.06 | 0.09 | 0.08 | 0.11 |
| **Luxembourg** | 0.03 | 0.02 | 0.03 | 0.05 | 0.08 | 0.05 | 0.07 |
| **Netherlands** | 0.01 | 0.02 | 0.03 | 0.08 | 0.12 | 0.06 | 0.08 |
| **Poland** | 0.01 | 0.02 | 0.03 | 0.09 | 0.14 | 0.12 | 0.16 |
| **Portugal** | 0.02 | 0.01 | 0.02 | 0.08 | 0.12 | 0.07 | 0.09 |
| **Romania** | 0.01 | 0.02 | 0.03 | 0.10 | 0.15 | 0.10 | 0.13 |
| **Slovakia** | 0.02 | 0.02 | 0.03 | 0.10 | 0.15 | 0.02 | 0.03 |
| **Slovenia** | 0.02 | 0.02 | 0.03 | 0.07 | 0.11 | 0.06 | 0.08 |
| **Spain** | 0.03 | 0.01 | 0.02 | 0.07 | 0.11 | 0.06 | 0.08 |
| **Sweden** | 0.03 | 0.01 | 0.02 | 0.05 | 0.07 | 0.02 | 0.03 |
| **UK** | 0.01 | 0.01 | 0.02 | 0.06 | 0.09 | 0.05 | 0.07 |
| **Europe-26** | 0.02 | 0.01 | 0.02 | 0.07 | 0.11 | 0.06 | 0.08 |
