# Supplementary material for: Reducing burden of disease from residential indoor air exposures in Europe (HEALTHVENT project)
Source: Environ Health. 2016 Mar 8;15(Suppl 1):35. doi: 10.1186/s12940-016-0101-8 (PMC4895703; doi:10.1186/s12940-016-0101-8)
Supplement: Additional file 3: — Peer review reports. (PDF 724 kb) [file 12940_2016_101_MOESM3_ESM.pdf]

Reviewer 1

Reducing burden of disease from residential indoor air exposures in Europe (HEALTHVENT project)

Manuscript: 1476-069X-14-S1-S6 (Healthy-Polis special issue)

Reviewer: James Milner

Recommendation: Revise

General comments

This paper attempts to understand optimal ventilation rates at the national level across 26 European countries and to contrast the benefits of optimizing ventilation with filtration of outdoor air pollution and control of indoor sources. This is an important topic given the key role of the housing sector in tackling both air pollution and climate change. Although the methods used are relatively simplistic, they are appropriate for analyses performed at the scale presented in the paper. The findings and conclusions are well supported by the results. I would therefore support publication. However, I believe the manuscript would benefit from a stronger statement of the aims of the work and some clarifications regarding the methods (see below).

My main concern relates to the assumption made by the authors that indoor-generated PM2.5 is equally as harmful to health as outdoor-generated PM2.5. There is little evidence to support this (though it is quite a commonly made assumption). I would like to see some form of sensitivity analysis in relation to this assumption, since it may have an impact on the final results (though probably not on the ultimate conclusions).

More detailed comments can be found below. I consider the suggested revisions to be relatively minor.

Specific comments

1. Is the question posed original, important and well defined?

The research question posed by the paper is important and I believe the methods used are appropriate to answer it. However, the aims of the paper are not currently well stated (in both the main text and in the abstract). The main statement of the aims comes towards the end of page 5, which states "This work aims to summarize the current understanding of the sources of health risks in indoor environments and their relationship to ventilation requirements". This doesn't really capture what the authors have actually done. There is a slightly better statement of the aims hidden in the Methods (top of page 7) but even this doesn't quite feel sufficient.

2. Are the data sound and well controlled?

Yes, although the source of some of the input data can be made clearer in the text, especially in relation to Table 2 (see below).

3. Is the interpretation (discussion and conclusion) well balanced and supported by the data?

Yes.

4. Are the methods appropriate and well described, and are sufficient details provided to allow others to evaluate and/or replicate the work?

The methods are generally well described. However, I have a few specific issues (see the additional comments below).

5. What are the strengths and weaknesses of the methods?

The methods are based on relatively simplistic tools (both the mass-balance exposure model and the risk/health impact model). However, I think they are appropriate for the type of analysis presented in the paper.

My main concern relates to the assumption of equal health risk associated with both outdoor- and indoor-generated PM2.5. The authors discuss this briefly in the Discussion section but this assumption is highly uncertain since almost all of the published epidemiology is based on outdoor PM2.5. The paper would benefit from greater emphasis on the sensitivity of the results to this assumption. Perhaps, the analysis (or part of it) could be repeated without the effects of indoor-generated PM2.5 or with a reduced exposure-response coefficient?

6. Can the writing, organization, tables and figures be improved?

Although the manuscript is generally well written, there are reoccurring problems, most importantly missing articles throughout the paper (e.g. a, the). For instance, in the abstract, "Based on measurements of the European Environment Agency (EEA),…" and "A framework for developing European health-based ventilation guidelines…" would be preferable. I realise that English is not the authors' first language (and, again, I should stress that most of the language used is very good). However, I think the paper would benefit from a thorough edit by a native English speaker.

Also, the figures appear to be quite pixelated and may not be in an appropriate format for publication.

7. When revisions are requested.

No preference.

8. Are there any ethical or competing interests issues you would like to raise?

None.

9. Additional comments

- I believe the abstract would benefit from a little editing. At present, the Background section feels unnecessarily long and there isn't enough detail in either the Methods or Results sections. The methods used for the health impact calculations aren't mentioned at all, despite being key to the paper. At the moment, I don't feel the main results are clearly stated in the abstract.

- The paper refers throughout to "optimization". I think a mathematician might object to the use of the term because the method doesn't actually use a formal optimization process. I don't think this is too much of a problem but I do think a clear statement of what the authors mean by optimization would be helpful (e.g. in the Methods). As I understand it, the "optimal" ventilation rate is just taken to be the one for which the health burden is smallest.

- I found some parts of the description of the risk model confusing, in particular towards the bottom of page 7. Firstly, the text states that the models are based on a predefined population attributable burden of disease for each exposure and disease. Is this simply referring to the calculation described later in the section (bottom of page 8)? Secondly, the text then states that "national estimates are then calculated from the national burden of disease data by scaling the attributable fraction according to the ratio of national versus European indoor concentration estimates for each pollutant". I'm afraid I could not follow the point being made here. Which "European" estimates are being referred to?

- On page 8 it is stated that "Traditional risk assessment methods estimate these [mortality and morbidity] separately as numbers of cases". I don't really agree with this statement. There are many health impact assessment methods which can consider both together and which are not incidence-based.

- The source of the data presented in Table 2 was not clear to me. Do these values relate to previous work by the authors? The source(s) used should be explained and appropriate references provided.

- The section describing the basis for the assumed source control levels (page 11) can be improved. Although the authors provide an explanation of the methods that can be used to achieve such source control for each pollutant, it is not clear how the specific reduction % was decided upon in each case. Are the reductions based on evidence or just plausible best guesses? For example, how do you know that implementing compulsory alarms will reduce CO sources by exactly 90%? The section would benefit from additional references to supporting literature.

- I do not understand why the source control scenario (scenario 3) did not also include an element of ventilation optimization. The start of the section describing the scenarios suggests that ventilation optimization would be "complemented" first by filtration and then by source control (page 9). This suggests that a ventilation optimization component would appear in all scenarios. Choosing just one "optimal" ventilation rate makes it difficult to ascertain the relative benefits due to ventilation and source control.

- There is a line on page 14 which states "substantial reductions have been proposed in the earlier work within the EU funded IAIAQ project". It is not clear what reductions are being referred to here. Is it reductions in the burden of disease due to indoor exposures? Why have reductions been proposed?

- The term "EU-26" is used throughout the paper and should be defined somewhere.

- There are two occasions where references are missing in the text (bottom of page 8 and below Table 3).

Reviewer 2

Dear Sotiris
Please find my detailed comments on the attachment and my review below:

The manuscript "Healthy-Polis 1476-069X-14-S1-S6" focusses on the calculation of the annual burden of disease caused by exposure to indoor air pollution.
This is very important work, given that the indoor environment is greatly ignored in relation to health.
The MS is generally well written and certainly merits publication.

Apart from some minor editing, as indicated in the attachment, my comments mainly aim to improve the reader's understanding. The areas requiring clarification are highlighted in the attachment together with some comments. The two most important ones are as follows:

1. Methods: My understanding is that the exposure analysis refers only to indoor exposure in the residential environment, without considering any time spent outdoors or in other indoor microenvironments; however, this is not clear in the text. Can you please clarify what the building stock represents.

2. The section on the "Risk model" directs the reader to several references of previous work. However, the reader may not be familiar with these methodologies and, most importantly, this significant part of the paper should stand alone. To enable a more friendly reading, the authors are kindly required to provide the data used in the methodology, step by step, as supplementary data (i.e. BoD, national estimates/statistics, PAF etc).This would improve a lot the quality and value of this publication.

Many thanks

Sani

Reducing burden of disease from residential indoor air exposures in Europe (HEALTHVENT project)

Arja Asikainen[1§], Paolo Carrer[2], Stylianos Kephalopoulos[3], Eduardo de Oliveira Fernandes[4], Pawel Wargocki[5], Otto Hänninen[1]

[1] National Institute for Health and Welfare, Department of health protection, Neulaniementie 4, 70210 Kuopio, Finland

[2] University of Milan, Department of Occupational Health, Via G.B. Grassi 74, 20157 Milan, Italy

[3] European Commission, Joint Research Centre, Via Enrico Fermi 2749, I - 21027 Ispra, Italy

[4] University of Porto, INEGI, Rua Dr. Roberto Frias, 4200 400 Porto, Portugal

[5] Technical University of Denmark, International Centre for Indoor Environment and Energy, DTU Civil Engineering, 2800 Kps. Lyngsby, Denmark

[§]Corresponding author

Email addresses:

AA: arja.asikainen@thl.fi

PC: paolo.carrer@unimi.it

SK: stylianos.kephalopoulos@jrc.ec.europa.eu

EOF: eof@fe.up.pt

PW: paw@byg.dtu.dk

OH: otto.hanninen@thl.fi

Abstract

Background

The annual burden of disease caused by inadequate indoor air quality is estimated to correspond to a loss of over 2 million healthy life years in the European Union (EU). This burden is caused by sources of indoor air pollution, including polluted outdoor air used to ventilate indoor spaces. Based on measurements of European Environment Agency (EEA), approximately 90% of EU citizens live in areas where the World Health Organization (WHO) guidelines for air quality for particulate matter sized < 2.5 mm ($PM_{2.5}$) are not met. ~~Because~~ sources of pollution reside in both indoor and outdoor air, selecting the most appropriate ventilation strategy to ensure that the health risks associated to exposure inside buildings are reduced is not simple and straightforward task.

Methods

Framework for developing European health-based ventilation guidelines was created in 2010-2013 in the EU-funded HEALTHVENT project. As a part of the project ~~potential of efficient~~ control policies to reduce the burden of disease caused by indoor exposures was estimated. ~~Analysis was based on scenario comparison using a model, which was based on mass-balance framework and changes in ventilation level~~.

Results

The quantitative comparison of three main policy approaches, (i) ~~changing~~ ventilation rates only; (ii) filtration of outdoor air; and (iii) indoor source control, showed that all three approaches are able to provide substantial reductions in the health risks varying from approximately 20% to 44%, corresponding to 400 000 and 900 000 saved healthy life years in EU-26.

Conclusions

Health effects of indoor air exposures can be decreased by increasing ventilation and the present modelling shows that controlling indoor air sources plays a major role when selecting appropriate ventilation rate. ~~In a case where~~ indoor sources cannot be removed or their emissions cannot be limited to an accepted level, ventilation needs to be increased to remove remaining pollutants. In these cases outdoor air pollution become the major source of pollution ~~in~~ indoors, and ~~it~~ needs to be taken into accou[illegible] Particulate matter, mainly coming from outdoors to indoors, is the main cause of health effects of indoor exposures in all European countries.

Background

In the period 2006-2010, focus on indoor air quality has been raised by WHO, who has issued specific guidelines addressing air exposure in indoor spaces [1, 2]. ~~Already during the previous~~ two decades WHO had coordinated systematic reviews of scientific evidence and set Air Quality Guidelines [3, 4] although not specific for indoor air.

Requirements for indoor air quality (IAQ) in buildings is prescribed by existing standards for ventilation, but are often poorly related on health. At present many ventilation standards (e.g. EN15251 [5]) define ventilation requirements in non-industrial buildings to meet comfort requirements of occupants, specified by the percentage of dissatisfied persons with indoor air quality and/or by the intensity of odour. While comfort is an important ~~outcome~~, it does not fully reflect more serious health impacts like asthma, allergies, chronic obstructive pulmonary disease, cardiovascular diseases, lung cancer and acute toxication that are caused by exposures to pollutants present in indoor air. There are no European guidelines ~~which~~ recommend how the buildings should be ventilated to reduce the health risks of the occupants' ~~exposed~~ to indoor air pollutants.

Direct scientific evidence on the relationship between ventilation and health is quite limited. Wargocki et al. (2002) [6] reviewed 105 papers published in peer-reviewed scientific journals, out of which 30 papers were judged to provide sufficient information on ventilation, health effects, data processing, and reporting. Ventilation was considered to be strongly associated with comfort (perceived air quality, PAQ) and health (including sick building syndrome (SBS) symptoms, inflammation, infections, asthma, allergy, and short-term sick leaves), and an association between ventilation and productivity (performance of office work) was indicated.

Similar results were obtained in the review by Seppänen et al. (2004) [7]. They concluded that the existing literature indicates that ventilation has a significant impact on several important human outcomes including: (1) communicable respiratory illnesses (disease prevalence or sick days); (2) sick building syndrome (SBS) symptoms; (3) task performance and productivity, and (4) perceived air quality (PAQ) as judged by building occupants or recruited sensory panels of assessors; and (5) respiratory allergies and asthma.

Li et al. (2007) [8] performed a systematic review of the role of the built environment in the transmission of airborne infectious agents. Specifically, they examined whether there was sufficiently strong evidence in the current literature to substantiate a contributory role of ventilation rates and airflow patterns in the airborne transmission of infectious agents in different indoor settings. They concluded that there is strong evidence ~~substantiating~~ the association between ventilation, air movements in buildings and the transmission/spread of infectious diseases such as measles, tuberculosis, chickenpox, influenza, smallpox and severe acute respiratory syndrome (SARS).

Sundell et al. (2011) [9] identified 27 papers published in peer reviewed journals providing sufficient information on both ventilation rates and health effects. Multiple health endpoints showed similar relationships with ventilation rate and were biologically plausible, although the literature did not provide clear evidence on particular agents. Higher ventilation rates in offices, up to about 25 l/s per person, were shown in the reviewed literature to be associated with reduced prevalence of SBS symptoms. Limited data suggested that inflammation, respiratory infections, asthma symptoms and short-term sick leave increase with lower ventilation rates. Home ventilation rates above 0.5 air changes per hour (h^{-1}) were shown in the reviewed papers to be associated with reduced risk of allergic manifestations among children in a Nordic climate.

None of the studies included in the reviews specifically addressed the role of outdoo air quality on indoor exposures, even though 90% of EU citizens live in areas where the WHO guidelines for air quality for $PM_{2.5}$ is not met [10]. Neither was the existence of indoor air sources systematically analysed nor exposure levels quantified or considered when associating ventilation and health. Therefore ~~the support~~ from these previous studies on determining the best combination of source control and ventilation levels ~~is limited~~. This work aims to summarize the current understanding of the sources of health risks in indoor environments and their relationship to ventilation requirements. The methods presented here allow for informed health-based optimization of efforts aimed at reducing harmful exposures and improving health of the occupants. The results are intended for development of national and international guidelines and standards, and can also be used as background information when analysing indoor air quality related issues in buildings.

Methods

Exposures

Ventilation plays a dual role in formation of indoor pollutant concentrations: on one hand it removes pollutants generated indoors from indoor spaces by ventilating the space with outdoor air, on the other hand, ventilation introduces outdoor air pollutants indoors [11, 12]. Assuming a constant outdoor pollution level and constant penetration efficiency, increasing ventilation directly leads to increased indoor exposures to outdoor pollutants. Even in the case of efficient filtering of particles in the intake air, detailed studies have shown that a substantial fraction of the outdoor air enters indoors via windows, doors, ventilation ducts, and cracks and leaks in the building envelope, leading to much lower actual filtration efficiency [13].

Due to the counter-acting roles of indoor and outdoor air sources on indoor exposures, a mass-balance model is needed to address it when defining prevailing indoor concentrations. A commonly used approach based on Dockery and Spengler [14] and adopted in Hänninen et al. [11], [15] is as follows:

Eq 1
$$\overline{C_i} = \frac{Pa}{a+k}\overline{C_a} + \frac{G}{V(a+k)} - \frac{\Delta C_i}{\Delta t(a+k)}$$

where C_i is the total indoor concentration (µg m^{-3}) of the pollutant in question, C_a is the concentration in the intake air, P is the probability of the pollutant remaining suspended after penetrating through the building envelope, α is air exchange rate (h^{-1}), k is the deposition rate of the pollutant indoors (h^{-1}), G is the indoor generation level (µg h^{-1}), V is the volume and t is the temperature of the indoor space. The third term covering the transient impacts of changing concentration can be considered zero for the sake of long-term average exposures. Detailed input data and more details of calculations are presented in Hänninen and Asikainen (2013) [16].

Because the aim of this study is to estimate how changes in ventilation affect exposures, the probability distributions of national ventilation rates in a building stock of year 2010 had to be estimated. Surprisingly limited data of measured ventilation rates are available for European countries. Due to this, available measured data was reviewed and a regression model was created combining the climatological and economical differences in the building stocks with ventilation rates. Further modelling with a Bayesian subjective probability approach was used for generation of lognormal probability distributions for ventilation rates in each EU-26 country (Table 1, method described in detailed elsewhere [17]).

Table 1.

Risk model

Large number of indoor air pollutants has been associated with health responses, but some of those either play a small role from the point of view of public health, or pose challenges for the exposure assessment or quantification of the burden of disease. Health determinants of housing in general are discussed in WHO 2011 [18], safe levels of specific chemicals indoors in WHO 2010 [2] and guidelines for exposure to dampness and mould specifically in WHO 2009 [1]. The current enhancement of the health impact assessment with the above described mass-balance approach to account for variable ventilation is built on the previous achievements of EnVIE [19] and IAIAQ projects [20] and the corresponding models for environmental burden of disease caused by indoor air quality. These models were based on a predefined population attributable burden of disease (BoD) for each exposure and disease and national estimates are then calculated from the national burden of disease data by scaling the attributable fraction according to the ratio of national versus European

indoor concentration estimates of each pollutant. (i.e. $PM_{2.5}$, outdoor bioaerosols, VOC, carbon oxide (CO) radon and dampness). In the current work the earlier $PM_{2.5}$, radon and dampness models were updated to the relative risk-based population attributable fraction (PAF) approach but keeping the IAIAQ disease classification. In addition, second hand smoke exposures at home were added using exposure data from a European survey [21].

Exposures to environmental pollutants are associated with increased mortality and morbidity. Traditional risk assessment methods estimate these separately as numbers of cases. The results from such incidence-based models are not comparable over different types of health endpoints and to improve comparability of impacts on various types of diseases and including mortality, disability adjusted life years (DALY) has been proposed as a common metric [22].

The burden of disease methodology makes the years of life lost (YLL) due to premature mortality and years lived with a disability (YLD) comparable and is summing them up as disability adjusted life years (DALY)

Eq 2 $$DALY = YLL + YLD$$

The disabilities caused by various types of diseases are calculated accounting for the duration of the disease (*L*) and scaled using a disease specific disability weight (*DW*):

Eq 3 $$YLD = DW \times L$$

In the current work the fraction of disease caused by the indoor exposures to various pollutants is estimated using national statistics on the overall background burden of the target diseases ~~(Table 1)~~ and calculating the population attributable fraction (*PAF*) as [**Error! Reference source not found.**]:

Eq 4 $$PAF = \frac{f \times (RR - 1)}{f \times (RR - 1) + 1}$$

where f is the fraction of population exposed to a given factor and RR is the relative risk of the exposed population. Now if the background burden of disease (BoD) is known the environmental burden of disease (EBD) caused by the current exposures (Table 2) can be calculated as

Eq 5 $$EBD = PAF \times BoD$$

The relative risk at the current exposure level can be estimated from epidemiological relative risk ($RR°$) expressed per a standard exposure increment, e.g. 10 µg m^{-3}:

Eq 6 $$RR = e^{(E \ln RR°)} = RR°^{E}$$

WHO estimates for national burden of disease in 2004 were used for the background BoD [24]. Pollutant specific diseases and methodology are presented in Table 3.

Table 2.

Table 3.

Exposure control scenarios

Three alternative exposure control scenarios were evaluated using the mass-balance enhanced burden of disease model to evaluate their efficiency to reduce BoD. The exposure control scenarios start from optimizing the ventilation rates only. As it proves inefficient, it is complemented firstly with control of filtration of outdoor pollutants and secondly with control of indoor sources.

Dilution by optimal ventilation

The first exposure reduction scenario is defined as finding the health-based optimum ventilation rate without any other actions that change indoor or outdoor sources. In this scenario the pollutant concentrations from indoor and outdoor sources compete so that pollutants from indoor sources are decreasing and pollutants from the outdoor sources are increasing when the ventilation rate is increased. The health-based

optimum level of ventilation is solved for each country among EU-26 by calculating burden of disease with ventilation rates from 0.1 to 50 lps pp.

The calculations assume that all indoor originating exposures follow the mass-balance dilution even though this is not self-evident for several indoor originating pollutants, especially radon, dampness, mould and carbon monoxide. Radon infiltrates typically from the soil below the buildings, and the infiltration may react to the under pressure indoors, which may increase in some ventilation systems at higher ventilation rates. Dampness may also be created by condensation and may thus increase at higher ventilation rates. Carbon monoxide is lethal at high exposure levels and more efficient dilution by higher ventilation may not be sufficient. However, for all these pollutants the benefits of higher ventilation rates are calculated assuming the mass-balance for a constant source term.

Filtration of intake air

Previous analyses of the sources of indoor exposures have shown that outdoor air is a significant source of exposure[illegible]herefore the second scenario was determined as an attempt to control the burden of disease by ~~filtration~~ the exposures originating from outdoor air. Because both ultrafine and coarse particles and chemically reactive pollutants like ozone have lower infiltration factors than $PM_{2.5}$, dominated by accumulation mode particles, the filtration was specified for $PM_{2.5}$ particles.

Three levels of filtration were compared. The baseline estimates assume that 90% of the outdoor $PM_{2.5}$ mass concentration penetrates indoors. In addition, realistic but increasingly challenging penetration levels of 70% and 50% were evaluated. These correspond to effective filtration of $PM_{2.5}$ mass concentration by 27% and 45%, respectively, filtration levels that can be achieved in real buildings at least when using mechanical ventilation systems [13]. When discussing the filtration efficiencies of filters and the above mentioned penetration efficiencies, it has to be noted that the

penetration efficiency is defined for the building, accounting for leaks and ventilation from windows, doors etc.

The health-based optimum ventilation was defined in this scenario also, and used when calculating the burden of disease results and the reduction potential compared to the baseline scenario.

Source control and minimum ventilation (4 lps pp)

The third approach to ~~optimizing~~ ventilation for health focuses first on indoor sources of exposures. Now, instead of attempting to dilute these sources as they are, they are first assumed to be controlled by other means as much as technically feasible before optimizing the ventilation for health. The assumed control potentials for the considered pollutants were:

- -90% for radon, carbon monoxide (CO) and second hand smoke (SHS)
- -50% for volatile organic compounds (VOC) and dampness
- -25% for particulate matter ($PM_{2.5}$)

These hypothetical source controls were defined to approach maximum technically feasible reductions. The radon estimate assumes efficient application and control of radon safe construction in radon-prone areas combined with control of second hand smoke exposures known to act synergistically with radon. Efficient second hand smoke reductions have already been demonstrated in Finland in both workplaces and in homes resulting a decrease in proportion of adolescents exposed to SHS from 17% in 1991 to 6% in 2009 [25] and the SHS policies are moving forward also on at European level. The carbon monoxide controls were aimed to be implemented by compulsory alarms that will allow for identification of malfunctioning devices before the risks occur.

VOC controls can be reached by comprehensive labelling systems for low emission products. Dampness controls need to combine structural improvements with

active/online and passive warning sensors. ~~The most challenging element was considered to be particulate matter.~~ The proposed 25% reduction can be achieved with target exhausts in kitchens, avoiding use of candles and improved design of combustion devices.

To provide some sensitivity analysis to estimate the effectiveness of source control, two other scenarios with lower and higher source control capabilities were also analysed. In the lower source control scenario (scenario 3.1) it was assumed a reduction of 80% for radon, CO and SHS and 25% reduction of $PM_{2.5}$, VOC and dampness exposures. In the higher source control scenario (scenario 3.2) a total control (100%) of radon, CO and SHS and 75% reduction of $PM_{2.5}$, VOC and dampness exposures were assumed.

In all source control scenarios the ventilation level was set to be 4 lps pp, which was defined as base ventilation rate in cases when ventilation must handle only human bio-effluent emissions (carbon dioxide (CO_2) and moisture) by work done in HealthVent to define the health based ventilation requirements [26].

Results

Attributable burden of disease in 2010

Exposures to indoor and outdoor originating pollutants were associated with a burden of disease corresponding to an annual loss of 2.1 million DALYs in EU-26. More than half of this burden (1.28 million DALYs) is caused by exposures to outdoor air pollution indoors. The remaining 0.74 million DALYs are associated with pollutants from various indoor sources.

The burden of disease caused by indoor exposures is dominated by cardiovascular (CV) diseases; 45% of the total burden comes from CV-diseases associated with outdoor particles, with an additional 12 % caused by indoor sources of exposures of

particles and second hand smoke (Figure 1). Cardiovascular diseases are followed by asthma (total of 12%) and lung cancer (23%). The remaining 8% is divided between various respiratory symptoms and conditions.

Figure 1.

The total burden of disease for individual countries varies between 2000 and 10 000 DALYs per million (Figure 2). The highest burden in Bulgaria is almost five times higher than that in Sweden. The higher levels in East-European countries are dominated by high contributions from outdoor sources. The contribution of outdoor sources varies between 46% (Ireland) to 75% (Bulgaria). The EU-26 average burden corresponds to slightly over 4000 DALY in a year per one million population.

Figure 2.

Source contributions to burden of disease

Overall in EU-26, over 50% of the total annual burden of disease associated with indoor exposures (4000 DALYs/million) is estimated to be caused by $PM_{2.5}$ originating from outdoor air, followed by particles from indoor sources, and radon (Figure 3).

The contribution of different sources to the total DALYs varies between countries. This can be seen when comparing the sources of the burden of disease in Finland (Figure 3) and in other EU-26 countries (Table 4) with the population-weighted mean of EU-26 countries. It is readily apparent that in Finland the role of ambient particles is lower than in Europe in general, but that both bio-aerosols (pollen) and radon play much more significant roles. Especially the contribution of radon is double to that of the European average, highlighting the geology peculiarities of the Finnish soil.

However, in Finland the burden of disease from lung cancer caused by radon exposures is alleviated partly by lower smoking prevalence. On average, 31% of over 15-year olds smoke daily or occasionally in EU countries and the smoking figures are lower only in Finland (25%), Sweden (25%) and Slovakia (22%) [21].
Dampness and mould problems continuously raise a lot of attention in Finland, too. Nevertheless, the burden of disease in Finland is from the lower end on the European scale (ranging from 1% to 11%), and only 3% is estimated to be caused by dampness in comparison with average of 5% in EU-26.

Figure 3.

Control scenario benefits including optimal ventilation

The burden of disease caused by indoor exposures, estimated above to be over 4 000 DALYs per year per a population of 1 million in EU-26, is significant. However, also substantial reductions have been proposed in the earlier work within the EU funded IAIAQ project [20]. The three alternative scenarios (and two additional source control scenarios) described earlier were tested to support policy development for controlling the risks and reducing the burden. The overall comparison of these scenarios in EU-26 is presented in Figure 4. The achievable health benefits were 20% for the dilution scenario, 38% for the filtration scenario, and 44% for the indoor source control scenario (changing from 41% to 54% depending on assumed source reductions).

Figure 4.

Each control scenario provides noteworthy health benefits. However, in the dilution-based scenario 1 the health benefits remain smallest because the reduction of indoor

originating exposures is compensated by infiltration of outdoor pollution when increasing ventilation rates. The European health optimum (lowest burden of disease achieved by changing national ventilation level) is found at mean ventilation level of 4.4 lps pp, which is clearly lower than the mean ventilation in the existing building stock (17 lps pp) defined by the regression modelled probability distributions (Table 1) [16, 17].

Approximately twice as high benefits are achievable by filtration of outdoor air in scenario 2. The results for maximum feasible filtration (with penetration fraction P = 50%) show that reduction in burden of disease approach 38 % or 800 000 DALYs in EU-26. The European optimum mean ventilation level is then 7.7 lps pp. Health-optimized ventilation level in addition to the filtration produces small additional improvements. This scenario would in practice imply substantial change towards mechanical ventilation systems in Europe. In the Nordic countries this is already the practice due to the energy efficiency norms, but in the majority of the European building stock the filtration scenario would require a substantial step towards installing mechanical systems.

However, largest health benefits can be achieved by the source control approach (scenario 3.0), which significantly reduces the need to control exposures by dilution. The benefits are approximately 44% from the baseline, or 940 000 DALYs in EU-26, and changing from reduction of 41% (865 000 DALY) with lower source control assumptions to 57% (1.21 million DALY) with higher source control assumptions, demonstrating source control to be more effective than dilution or filtration even with smaller reductions of source exposures.

In addition to ~~of~~ higher health benefits~~, in comparison with~~ the filtration-based scenario 2 the advantage ~~is that with source control the lower dilution need~~ (i.e.

enabling lower ventilation rate) allow also for lower infiltration of outdoor particles and therefore the feasibility of ~~the~~ approach is better in the current building stock. Moreover, ~~with~~ lower ventilation rates ~~require~~ the source control approach is likely to prove also more energy-efficient.

Further analysis of the contribution of indoor and outdoor sources ~~on these scenarios~~ shows, that with the dilution scenario the health benefits are not only due to smaller proportion of the indoor contribution (i.e. the dilution of the pollutants from the indoor sources) but ~~is mainly based on~~ the lower ventilation rates that ~~actually~~ limit~~s~~ the penetration of the outdoor pollutants ~~to~~ indoors.

In the filtration scenario the health benefits are due to filtration of the outdoor pollutants and also effective dilution of the indoor pollutants as the health-based optimal ventilation levels are higher.

~~Also~~ in the source control scenarios the health benefits are a result of both effects~~;~~ ~~firstly by the~~ lower indoor sources due to the source control and ~~secondly by~~ lower penetration of outdoor pollutants due to low level of ventilation.

Discussion

The results suggest that (i) there is a substantial burden of disease associated with exposures through inhalation taking place indoors and that (ii) these risks can substantially be reduced by various policies that include a range of control actions affecting indoor pollution sources, infiltration of outdoor pollutants, and ventilation levels. Besides the estimated health benefits and policy implementation costs, the suggested prioritization of the policies depends also on the uncertainties of the estimates.

Model uncertainties are causing the ~~largest~~ concerns ~~here~~. They include the selection of pollutants and health end-points associated with them. It is clear that in the current

context a substantial uncertainty is raised from here: it is not clear how much the burden of disease estimates are underestimated due to the dozens of ignored exposures or missing health endpoints for the included exposures. At best the model uncertainties can and should be qualitatively judged by experts before the general implementation of results.

Parameter uncertainties are easiest to estimate and evaluate. Quantification of the exposure-response relationships and mass-balance model for exposures belong also to the parameter uncertainties. Variable degree of uncertainty exists in the exposure-response response relationships based on epidemiological studies. For some of the included pollutants, like $PM_{2.5}$ originating from outdoor air, this data is based on a large number of studies, representing very large populations in different climatological regions. The exposure-response relationship of ambient particles has also been used for indoor generated particles. The indoor generated particles have partly similar composition, originating from combustion processes or being re-suspended particles originating from soil, for which it is reasonable to assume similar toxicity as for the ambient particles. Some particle fractions, especially the particles from cooking of food, from the occupants, and from textiles, have a different chemical composition with limited direct evidence on their toxicity.

Scenario uncertainties are inherent for any future forecasts; we may not know all changes in the systems under investigation and therefore must rely on assumptions. When selecting policies for implementation, the implementation timeframe should also be considered. Most significant element in the scenario uncertainties is related to the development of future building stocks. The current ventilation guidelines are intentionally formulated so that the focus is on the key parameters in terms of health, the exposures, and there are as little as possible elements that require specific

technical solutions. An example of such an issue is the filtration of outdoor air pollution, especially $PM_{2.5}$, but also pollen, other biological particles, ozone, ultrafine traffic particles and so on. Infiltration of ambient particles depends on air exchange rates, size distribution of the outdoor particles, and filtration of the intake air. At lower air exchange rates the prolonged residence time of air indoors and corresponding deposition of particles on indoor surfaces reduces indoor exposures even when the outdoor air is not filtrated. Using window frames and other sedimentation chambers allows for filtrating particles even in natural ventilation systems. Nevertheless, active filtration becomes efficient only in mechanical systems using high quality (above FP7) filters.

The used ventilation rate estimates per occupant (lps pp) are calculated using average residence sizes and average numbers of occupants in each country. Population weighted average outdoor concentrations have also been used in estimating the indoor exposures. It is clear that the air filtration needs for a specific building have to be defined using the ambient air quality at the selected building location. In all countries considered there are locations where the outdoor levels exceed the WHO guidelines much more than the national averages used here indicate. When the current methods proposed for determining the potential filtration needs, they have to be applied with worst case estimates for the actual building site, accounting for the whole service life cycle.

Largest health benefits were projected for the source control policies. It is obvious that the benefits are achievable only if the source controls work as efficiently as proposed and that the efficiency of the source controls must be confirmed with follow-up (e.g. auditing) of exposure levels after the policy enforcement.

Conclusions

Over 2 million disability adjusted life years (DALY) are annually lost in the European Union due to compromised indoor air quality, but this burden of disease can be reduced by adjusting ventilation, filtration of intake air and by controlling indoor sources. All three approaches are able to provide substantial reductions in the health risks from approximately 20 % to almost 50%, corresponding to 400 000 and 900 000 saved DALYs in EU-26. Thus selection of strategies has substantial impact on the expected benefits.

The projected health benefits can be achieved if the controls on ventilation and sources are fully implemented as defined in the scenario descriptions. In the case of selecting some of the proposed strategies for implementation, a careful follow-up plan has to be developed for ensuring that the controls are effective and match the requirements of the benefit calculations.

List of abbreviations used

BoD = burden of disease

CO = carbon monoxide

CO2 = carbon dioxide

CV = cardiovascular

DALY = disability adjusted life years

DW = disability weight

EBD = environmental burden of disease

EU = European Union

IAQ = indoor air quality

L = duration of the disease

lps pp = liters per second per person

PAF = population attributable fraction

PAQ = perceived air quality

PM2.5 = particulate matter sized < 2.5 mm

RR = relative risk

SARS = severe acute respiratory syndrome

SBS = sick building syndrome

SHS = second hand smoke

VOC = volatile organic compounds

WHO = World Health Organization

YLD = years lived with a disability

YLL = years of life lost

Competing interests

The authors declare that they have no competing interests.

Authors' contributions

AA carried out the modelling and calculation of results and drafted the manuscript. PC participated on the literature review for the background section and in the design of the study. EOF participated in the design of the study. SK participated in the design of the study and helped to draft the manuscript. PW participated in the design of the study, coordination and helped to draft the manuscript. OH participated on the design of the calculations, coordination and helped to draft the manuscript. All authors read and approved the final manuscript.

Funding

This work has been supported by the EU Health Programme HEALTHVENT Grant Nr. 2009 12 08, EU FP7 (TRANSPHORM), Academy of Finland (PM Sizex) and Ministry of Social Affairs and Health project TEKAISU.

References

1. WHO: *Dampness and mould. WHO Guidelines for Indoor Air Quality*. WHO Regional office for Europe, Copenhagen 2009, ISBN 798 92 890 4168 3. http://www.euro.who.int/document/E92645.pdf (accessed 2014-08-22).
2. WHO: *Guidelines for Indoor Air Quality: Selected pollutants*. World Health Organization, Regional Office for Europe, Copenhagen 2010, Denmark. ISBN 978 92 890 0213 4.
3. WHO: *Air quality guidelines for Europe*. World Health Organization, Regional Office for Europe, European series, No 23, Copenhagen 1987, Denmark
4. WHO: *Air quality guidelines for Europe; Second edition*. World Health Organization, Regional Office for Europe, No 91, Copenhagen 2000, Denmark. http://www.euro.who.int/document/e71922.pdf (accessed 2014-08-22).
   http://www.euro.who.int/__data/assets/pdf_file/0009/128169/e94535.pdf (accessed 2014-09-22
5. EN15251: *CEN Standard on Indoor environmental input parameters for design and assessment of energy performance of buildings- addressing indoor air quality, thermal environment, lighting and acoustics*. European Committee For Standardization (CEN) 2007, Brussels. 52 pp
6. Wargocki P, Sundell J, Bischof W, Brundrett G, Fanger PO, Gyntelberg F, et al: **Ventilation and health in non-industrial indoor environments: report from a European multidisciplinary scientific consensus meeting (EUROVEN).** *Indoor Air* 2002, **12:** 113-128

7. Seppänen OA, Fisk WJ: **Summary of human responses to ventilation**. *Indoor Air* 2004, 14 (Suppl 7):102–118
8. Li Y, Leung GM, Tang JW, Yang X, Chao CYH, Lin JZ, Lu JW, Nielsen PV, Niu J, Qian H, Sleigh AC, Su H-JJ, Sundell J, Wong TW, Yuen PL. **Role of ventilation in airborne transmission of infectious agents in the built environment – a multidisciplinary systematic review.** *Indoor Air* 2007, **17**: 2–18
9. Sundell J, Levin H, Nazaroff WW, Cain WS, Fisk WJ, Grimsrud DT, Gyntelberg F, Li Y, Persily AK, Pickering AC, Samet JM, Spengler JD, Taylor ST, Weschler CJ: **Ventilation rates and health: multidisciplinary review of the scientific literature**. *Indoor Air* 2011, **21**: 191-204
10. EEA: *Air quality in Europe*. European Environment Agency Report 9/2013, 2013, 112 pp. ISSN 1725-9177 http://www.eea.europa.eu/publications/air-quality-in-europe-2013/at_download/file (accessed 2015-01-30)
11. Hänninen O, Lebret E, Ilacqua V, Katsouyanni K, Künzli N: **Infiltration of ambient $PM_{2.5}$ and levels of indoor generated non-ETS $PM_{2.5}$ in residences of four European cities**. *Atmos. Environ.* 2004, **38**: 6411-6423
12. Hänninen OO, Palonen J, Tuomisto JT, Yli-Tuomi T, Seppänen O, Jantunen MJ, et al.: **Reduction potential of urban $PM_{2.5}$ mortality risk using modern ventilation systems in buildings.** *Indoor Air* 2005, **15:** 246-256 doi:10.1111/j.1600-0668.2005.00365.x
13. Fisk WJ, Faulkner D, Palonen J, Seppänen O: **Performance and costs of particle air filtration technologies**. Indoor Air 2002, **12**: 223-234

14. Dockery DW, Spengler JD: **Personal exposure to respirable particulates and sulfates**. *J. Air Pollut. Control Assoc*. 1981, **31:** 153-159

15. Hänninen O, Sorjamaa R, Lipponen P, Cyrys J, Lanki T, Pekkanen J: **Aerosol-based modelling of infiltration of ambient $PM_{2.5}$ and evaluation against population-based measurements in homes in Helsinki, Finland**. *J. Aerosol Sci*. 2013, **66**:111-122

16. Hänninen O, Asikainen A (eds.): *Efficient reduction of indoor exposures: Health benefits from optimizing ventilation, filtration and indoor source controls*. National Institute for Health and Welfare (THL). Report 2/2013. Helsinki 2013. ISBN 978-952-245-821-6 (printed) ISBN 978-952-245-822-3 (online publication) http://urn.fi/URN:ISBN:978-952-245-822-3

17. Asikainen A, Hänninen O, Brelih N, Bischof W, Hartmann T, Carrer P, Wargocki P: **Proportion of residences in European countries with ventilation rates below the regulation based limit value**. *Int J. Vent*. 2013, **12**: 129-134

18. WHO: *Environmental burden of disease associated with inadequate housing*. World Health Organization, Regional Office for Europe, Copenhagen 2011, Denmark http://www.euro.who.int/__data/assets/pdf_file/0003/142077/e95004.pdf (accessed 2014-08-22)

19. Oliveira Fernandes de E, Jantunen M, Carrer P, Seppänen O, Harrison P, Kephalopoulos S: *Co-ordination Action on Indoor Air Quality and Health Effects (ENVIE).* Final report, 2009, http://paginas.fe.up.pt/~envie/documents/finalreports/Final%20Reports%2

0Publishable/Publishable%20final%20activity%20report.pdf (accessed 2014-08-22)

20. Jantunen M, de Oliveira Fernandes E, Carrer P, Kephalopoulos S: *Promoting actions for healthy indoor air (IAIAQ).* European Commission Directorate General for Health and Consumers. Luxembourg 2011. ISBN 978-92-79-20419-7. http://ec.europa.eu/health/healthy_environments/docs/env_iaiaq.pdf (accessed 2014-08-22)
21. EC: *Survey on Tobacco*. Analytical report. European Commission Flash Eurobarometer 253, 2009 http://ec.europa.eu/public_opinion/flash/fl_253_en.pdf (accessed 2014-08-22).
22. Murray CJ, Lopez AD: **Alternative projections of mortality and disability by cause 1990-2020: Global Burden of Disease Study**. *Lancet* 1997, **349:** 1498-1504. doi:10.1016/S0140-6736(96)07492-2
23. Hänninen O, Knol A (eds.): *European perspectives on Environmental Burden of Disease; Estimates for nine stressors in six countries.* THL Reports 1/2011, Helsinki 2011, Finland. ISBN 978-952- 245-413-3. http://urn.fi/URN:NBN:fi-fe201205085022 (accessed 2015-02-12)
24. WHO: *The global burden of disease 2004 update*. World Health Organization, Regional Office for Europe, 2008, Switzerland http://www.who.int/healthinfo/global_burden_disease/GBD_report_2004update_full.pdf
25. Raisamo SU, Doku DT, Heloma A, Rimpelä AH: **Persistence of socioeconomic differences in adolescents' environmental tobacco**

smoke exposure in Finland: 1991–2009. *Scandinavian Journal of Public Health* 2014, **42**: 184–193.

26. ECA: *Guidelines for health-based ventilation in Europe (HealthVent).* Report nr 30 of European Collaborative Action (ECA), Urban Air, Indoor Environment And Human Exposure. Environment and Quality of Life, EC Joint Research Centre 2015, Ispra, Italy
27. Pope CA, Burnett RT, Thun MJ, Calle EE, Krewski D, Ito K, et al.: **Lung cancer, cardiopulmonary mortality, and long-term exposure to fine particulate air pollution**. *JAMA* 2002, **287**: 1132-1141
28. Darby S, Hill D, Auvinen A, BarrosDios JM, Baysson H, Bochicchio F, Deo H, et al.: **Radon in homes and lung cancer risk: collaborative analysis of individual data from 13 European case control studies.** *British Medical J*. 2005, **330**: 223–226
29. Fisk WJ, Lei-Gomez Q, Mendell MJ: **Meta-analyses of the associations of respiratory health effects with dampness and mold in homes**. *Indoor Air* 2007, **17**: 284-296
30. US Surgeon General: *The health consequences of involuntary exposure to tobacco smoke*. A Report of the Surgeon General Office on Smoking and Health, Centers for Disease Control and Prevention, Atlanta 2006, US http://www.ncbi.nlm.nih.gov/books/NBK44324/ (accessed 2015-02-12)
31. Jaakkola MS, Piipari R, et al.: **Environmental tobacco smoke and adult-onset asthma: a population-based incident case-control study**. *Am J Public Health* 2003, **93**: 2055-60

Figure legends

Figure 1. Attributable burden of diseases due to indoor exposures in 2010 in EU-26. The lighter shade represents the maximum fraction that can be reduced by actions (scenarios) presented in this paper

Figure 2. Total burden of disease as DALY/million population from indoor exposures in EU-26 countries with division to indoor and outdoor sources in the 2010 building stock

Figure 3. Burden of disease attributable to indoor exposures in EU-26 (2.1 M DALY/a) and in Finland (13 k DALY/a) in 2010 divided into source contributions.

Figure 4. Burden of disease at the baseline (2010) in comparison with alternative potential control strategies in EU-26 (in millions of DALYs).

Tables

Table 1. Estimated ventilation rate distributions in European countries [17].

	Air exchange rate			Ventilation rate per occupant		
Country	Mean	Median	One-GSD range[a]	Mean	Median	One-GSD range[a]
	h^{-1}	h^{-1}	h^{-1}	lps pp	lps pp	lps pp
**Austria**	0,9	0,7	(0.4-1.3)	25	21	(11.1-39.1)
**Belgium**	0,7	0,6	(0.3-1.1)	17	14	(7.6-26.7)
**Bulgaria**	0,7	0,6	(0.3-1.1)	15	12	(6.4-22.3)
**Cyprus**	1,2	1,0	(0.5-1.9)	24	20	(10.6-37.2)
**Czech Republic**	0,6	0,5	(0.3-1.0)	14	11	(6.0-21.1)
**Denmark**	0,7	0,5	(0.3-1.0)	24	20	(10.4-36.6)
**Estonia**	0,7	0,5	(0.3-1.0)	13	10	(5.5-19.4)
**Finland**	0,7	0,5	(0.3-1.0)	17	14	(7.5-26.3)
**France**	0,6	0,5	(0.3-1.0)	18	14	(7.7-27.1)
**Germany**	0,7	0,6	(0.3-1.0)	20	17	(8.8-31.0)
**Greece**	1,0	0,8	(0.4-1.5)	20	17	(8.8-30.9)
**Hungary**	0,8	0,6	(0.3-1.2)	16	13	(6.8-24.0)
**Ireland**	0,6	0,5	(0.3-0.9)	14	12	(6.2-21.9)
**Italy**	0,8	0,6	(0.3-1.2)	21	17	(9.2-32.4)
**Latvia**	0,7	0,5	(0.3-1.0)	11	9	(4.9-17.2)
**Lithuania**	0,7	0,6	(0.3-1.0)	11	9	(4.9-17.3)
**Luxembourg**	0,9	0,7	(0.4-1.3)	32	26	(14.1-49.5)
**Netherlands**	0,7	0,6	(0.3-1.0)	21	17	(9.1-32.1)
**Poland**	0,7	0,6	(0.3-1.1)	11	9	(4.8-16.7)
**Portugal**	0,7	0,6	(0.3-1.1)	15	12	(6.6-23.1)
**Romania**	0,8	0,6	(0.3-1.2)	7	6	(3.2-11.1)
**Slovakia**	0,8	0,6	(0.3-1.2)	12	10	(5.1-17.9)
**Slovenia**	0,7	0,6	(0.3-1.1)	13	11	(5.9-20.7)
**Spain**	0,8	0,7	(0.3-1.2)	20	17	(8.9-31.3)
**Sweden**	0,6	0,5	(0.3-1.0)	20	17	(9.0-31.5)
**UK**	0,6	0,5	(0.3-0.9)	15	13	(6.8-23.8)
**EU-26**	**0,7**	**0,6**	(0.3-1.1)	**17**	**14**	(7.3-25.6)

Table 2. Outdoor and indoor exposure levels ($PM_{2.5}$, radon and VOC) and prevalence of exposure (dampness in homes and second hand smoke of non-smoking population) in European countries used for burden of disease calculations.

	**Out. $PM_{2.5}$**	**Ind. $PM_{2.5}$**	**Out. VOC**	**Ind. VOC**	**Ind. Radon**	**Dampness homes**	**SHS non-smokers**
	$\mu g\ m^{-3}$	$\mu g\ m^{-3}$	$\mu g\ m^{-3}$	$\mu g\ m^{-3}$	$Bq\ m^{-3}$	%	%
**Austria**	17	5	103	298	97	8	14
**Belgium**	19	5	103	298	69	14	18
**Bulgaria**	22	5	103	298	30	n/a	23
**Cyprus**	23	4	103	298	7	30	31
**Czech Republic**	23	5	116	334	140	16	16
**Denmark**	13	3	103	298	53	11	17
**Estonia**	11	3	103	298	120	23	16
**Finland**	9	3	64	226	120	5	2
**France**	12	5	77	223	89	14	9
**Germany**	16	5	103	297	50	13	13
**Greece**	21	4	155	345	55	19	28
**Hungary**	25	5	103	298	107	19	12
**Ireland**	8	3	103	298	89	15	14
**Italy**	20	4	181	489	70	21	11
**Latvia**	12	3	103	298	0	26	12
**Lithuania**	14	3	103	298	55	25	28
**Luxembourg**	12	5	52	148	115	15	8
**Netherlands**	19	5	46	134	30	18	15
**Poland**	22	5	103	298	49	37	21
**Portugal**	18	4	38	213	86	20	13
**Romania**	23	5	103	298	45	29	23
**Slovakia**	23	5	103	298	87	6	13
**Slovenia**	17	5	103	298	87	17	14
**Spain**	16	4	103	298	90	18	20
**Sweden**	10	3	77	223	108	6	3
**UK**	13	3	85	245	20	15	7
**EU-26**	**17**	**4**	**104**	**297**	**64**	**18**	**14**

Table 3. Diseases and exposure-response relationships included in this assessment.

Exposures[a]	Health endpoints	WHO	RR	PAF	RR & PAF source(s)	BoD calculation[b]
**$PM_{2.5}$**	Asthma	W113	–[c]	f(RR, E)[d]	Pope et al. 2002	PAF(E, RR) × BoD_{2004}
	Lung cancer	W067	–[c]	f(RR, E)[d]	Pope et al. 2002	PAF(E, RR) × BoD_{2004}
	CV-diseases	W104	–[c]	f(RR, E)[d]	Pope et al. 2002	PAF(E, RR) × BoD_{2004}
	COPD	W112	–[c]	f(RR, E)[d]	Pope et al. 2002	PAF(E, RR) × BoD_{2004}
**Outdoor bioaerosols**	Asthma	W113	–[c]	0.1[e]	Jantunen et al., 2010 [20]	PAF × BoD_{2004}
**VOC**	Asthma	W113	–[c]	0.05	Jantunen et al., 2010 [20]	C/C_{EU} × PAF × BoD_{2004}
**CO**	Acute toxication caused by carbon monoxide	n/a	–[c]	0.9	Jantunen et al., 2010 [20]	Cases x 20 years lost/case
**Radon**	Lung cancer	W067	0.0014	f(RR, E)[d]	Darby et al., 2005 [	PAF(E, RR) × BoD_{2004}
**Home dampness**	Respiratory infections	W038	1.37	f(RR, E)[d]	Fisk et al., 2007	PAF(E, RR) × BoD_{2004}
	Asthma	W113	1.5	f(RR, E)[d]	Fisk et al., 2007	PAF(E, RR) × BoD_{2004}
**SHS[f]**	Lung cancer	W067	1.21	f(RR, E)[d]	US S.G. 2006	PAF(E, RR) × BoD_{2004}
	Ischaemic heart disease	W107	1.27	f(RR, E)[d]	US S.G. 2006	PAF(E, RR) × BoD_{2004}
	Asthma	W113	1.97	f(RR, E)[d]	Jaakkola et al., 2003	PAF(E, RR) × BoD_{2004}

[a] Population weighted average in EU26

[b] C = National population weighted concentration, CEU = European average concentration, E = National population weighted exposure

[c] Expert judgment PAF from the EnVIE panel used directly [19], see column PAF

[d] Calculated as PAF=(f×(RR-1))/((f×(RR-1))+1), where RR = RR°E [**Error! Reference source not found.**].

[e] Original value of 0.25 in Jantunen et al. (2010) [20] adjusted to 0.1 due to separation of indoor and outdoor sources and focusing on pollen from outdoor air

[f] Second hand smoke exposure of non-smoking adults at home.

Table 4. Contribution (%) of different sources to the total DALYs in 2010.

	Ind. $PM_{2.5}$	Radon	Ind. VOC	CO	Damp.	SHS	Out. $PM_{2.5}$	Bio-aeros ols	Out. VOC
**Austria**	18	11	1	1	1	5	58	3	0
**Belgium**	18	9	1	0	4	4	60	3	0
**Bulgaria**	17	2	0	0	3	2	74	1	0
**Cyprus**	11	0	1	0	11	12	61	3	0
**Czech Republic**	14	12	1	4	3	3	61	2	0
**Denmark**	13	9	2	3	3	6	59	5	1
**Estonia**	14	13	1	5	8	4	52	3	0
**Finland**	16	16	2	4	3	2	50	7	1
**France**	20	18	2	0	5	2	46	6	1
**Germany**	20	6	1	1	3	4	60	3	0
**Greece**	14	6	1	1	4	5	68	2	0
**Hungary**	15	12	0	1	1	1	69	1	0
**Ireland**	13	12	4	2	11	12	34	11	1
**Italy**	14	9	2	1	4	3	64	3	1
**Latvia**	15	6	1	2	7	3	64	2	0
**Lithuania**	14	5	0	0	6	10	63	1	0
**Luxembourg**	21	15	1	1	6	3	47	5	0
**Netherlands**	18	4	1	1	6	5	61	5	0
**Poland**	15	5	1	1	6	3	66	2	0
**Portugal**	14	7	1	1	7	4	62	4	0
**Romania**	16	3	0	1	7	2	70	1	0
**Slovakia**	16	7	1	1	2	3	70	2	0
**Slovenia**	17	11	1	2	5	3	56	3	0
**Spain**	14	14	1	0	5	4	57	4	0
**Sweden**	16	15	2	2	3	3	54	6	1
**United**	13	3	2	1	8	5	59	8	1
**EU-26**	**16**	**8**	**1**	**1**	**5**	**4**	**62**	**3**	**0**
